# Multi-omics analysis reveals Protein Kinase A-associated regulatory remodeling during adaptation of *Trichoderma reesei* to lignocellulosic substrate

**DOI:** 10.1007/s11274-026-05252-x

**Published:** 2026-09-08

**Authors:** Hermano Zenaide-Neto, Wellington Ramos Pedersoli, David Batista Maués, Lucas Matheus Soares Pereira, Iasmin Cartaxo Taveira, Thiago de Andrade Simon, Andrei S. Steindorff, Vitor Marcel Faça, Renato Graciano de Paula, Roberto N. Silva

**Affiliations:** 1 Department of Biochemistry and Immunology, Molecular Biotechnology Laboratory, Ribeirão Preto Medical School (FMRP), University of São Paulo, Ribeirão Preto, SP 14049-900 Brazil; 2 Lawrence Berkeley National Laboratory, Joint Genome Institute, Berkeley, CA 94720 USA; 3 Department of Biochemistry and Immunology, Cancer Proteomics Laboratory, Ribeirão Preto Medical School (FMRP), University of São Paulo, Ribeirão Preto, SP 14049-900 Brazil; 4 Department of Physiological Sciences, Health Sciences Center, Laboratory of Structural and Functional Biochemistry, Federal University of Espírito Santo, Vitória, ES 29047-105 Brazil; 5 National Institute of Science and Technology in Human Pathogenic Fungi, Ribeirão Preto, Brazil

**Keywords:** Carbon-source adaptation, Multi-omics, Phosphoproteomics, PKAc1, Sugarcane bagasse, *Trichoderma reesei*

## Abstract

The filamentous fungus *Trichoderma reesei* is a major industrial source of holocellulolytic enzymes, and its response to complex carbon sources is regulated by nutrient-sensing mechanisms, including the cyclic adenosine monophosphate (cAMP)–protein kinase A (PKA) signaling pathway. Here, we integrated transcriptomics, quantitative proteomics, and phosphoproteomics to analyze PKAc1-associated responses in the parental strain QM9414 and a Δ*pkac1* strain cultivated under glucose or sugarcane bagasse conditions. Deletion of *pkac1* was associated with altered growth-related phenotypes and reduced extracellular activities of selected biomass-depolymerizing enzymes. Multi-omics profiling revealed condition-dependent changes affecting subsets of carbohydrate-active enzymes (CAZymes) genes and proteins, nutrient transporters, stress-associated proteins, and regulatory factors. Phosphoproteomics identified phosphorylation-state changes associated with *pkac1* deletion, including reduced phosphorylation at sites enriched for the PKA consensus motif. In silico peptide docking was used to prioritize candidate PKAc1-associated substrates for future validation, including a Sec 7-derived peptide with favorable docking behavior relative to the control peptide. Together, these data support a working model in which PKAc1 contributes to regulatory and phosphorylation-state remodeling during adaptation to sugarcane bagasse, with effects on the magnitude and/or timing of selected CAZyme-related outputs in *T. reesei*.

**Supplementary Information:**

The online version contains supplementary material available at https://doi.org/10.1007/s11274-026-05252-x.

## Introduction

Lignocellulosic biofuels offer a sustainable alternative to fossil fuels, but the efficient saccharification of plant biomass remains a technical challenge due to biomass recalcitrance (IEA 2025). Second-generation (cellulosic) ethanol production requires robust enzyme systems to deconstruct cellulose and hemicellulose into fermentable sugars. The filamentous fungus *Trichoderma reesei* is the primary industrial source of these cellulases and hemicellulases, due to its exceptional secretion capacity developed over decades of strain improvement (Himmel et al. 2007). However, the native production of lignocellulolytic enzymes in *T. reesei* is tightly regulated by nutrient availability, catabolite repression mechanisms and environmental signals such as light (Schmoll 2018), particularly the Cre1-mediated repression that modulates responses even to strong inducers like sophorose (Antoniêto et al. 2016). Efficient enzyme expression is repressed in the presence of simpler carbon sources (e.g., glucose) and induced only when the fungus detects complex polysaccharides such as cellulose (Bischof et al. 2016; Yan et al. 2021), triggering a metabolic reconfiguration distinct from glucose catabolism (Dos Santos Castro et al. 2014a). This regulation is mediated by a complex network of transcription factors, such as the master regulator Xyr1 (Dos Santos Castro et al. 2016) and Cre1 (Antoniêto et al. 2016), and signaling pathways that sense the carbon source: the cAMP pathway, which is crucial for secretion (Nogueira et al. 2015), the MAPK pathway, which plays a dual role in metabolism and cellulase control (de Paula et al. 2018; Schalamun et al. 2023), and vacuolar calcium transport systems that coordinate growth signaling with production (Oshiquiri et al. 2024).

Among the key signaling pathways, the cyclic AMP-dependent protein kinase A (PKA) pathway is a major regulator of nutrient-responsive signaling in fungi and influences hydrolase expression downstream of carbon sensing (Baker 2018; Schuster et al. 2012). PKA acts downstream of glucose sensing systems and coordinates growth, development, and metabolism according to nutrient status (Druzhinina and Kubicek 2017; Budovskaya et al. 2005). In model yeasts and filamentous fungi, PKA activity influences a wide range of processes, from sporulation and stress responses to the expression of hydrolytic enzymes (Budovskaya et al. 2005; Howard et al. 2001). Previous studies in *Neurospora crassa* and *Aspergillus fumigatus* demonstrated that PKA mutants exhibit reduced growth and developmental abnormalities (Grosse et al. 2008; Banno et al. 2005). In *T. reesei*, indirect evidence has hinted at PKA’s involvement in cellulase regulation and secondary metabolism (Hinterdobler et al. 2019). Consistent with this, the elevation of cAMP was shown to activate calcium signaling and boost cellulase gene expression (Chen et al. 2021), and phosphoproteomic analysis of *T. reesei* suggested PKA might post-translationally regulate secreted enzymes (Pedersoli et al. 2021). Moreover, the deletion of the PKA catalytic subunit (*pkac1*) in *T. reesei* (strain QM6a) led to impaired cellulase production and growth defects (Li et al. 2022). Although PKA/PKAc1 has previously been implicated in growth, cellulase production, light-dependent regulation, and secondary metabolism in *T. reesei*, its effects across transcriptomic, proteomic, and phosphoproteomic layers have not been examined in an integrated experimental framework.

In this work, we used an integrated multi-omics approach to examine PKAc1-associated regulatory responses in *T. reesei* QM9414 during adaptation to sugarcane bagasse. In our work, sugarcane bagasse was selected because it represents an industrially relevant lignocellulosic substrate composed of cellulose, hemicellulose, lignin, and associated minor components. Unlike purified cellulose, this complex biomass matrix requires broader physiological adaptation involving polysaccharide sensing, transport, enzyme secretion, and stress-associated responses. Therefore, the responses described here should be interpreted as adaptation to a complex lignocellulosic substrate rather than as cellulose-specific effects. We characterized a Δ*pkac1* strain and compared it with the parental strain under carbon-repressing (glucose) and lignocellulose-associated (sugarcane bagasse) substrates. Using RNA-seq, quantitative proteomics, and phosphoproteomics, we profiled changes in gene expression, protein abundance, and phosphorylation states associated with pkac1 deletion. In addition, we performed motif analysis and in silico peptide docking to prioritize candidate PKAc1-associated substrates for future validation. This study presents a systems-level view of PKAc1-associated remodeling during growth on a complex lignocellulosic substrate, while distinguishing retained induction from altered response amplitude and downstream regulatory outputs.

## Materials and methods

### Strains and cultivation

The parental *T. reesei* strain used in this study was QM9414 (ATCC26921), and the Δ*pkac1* strain was obtained from the previously described *pkac1* deletion background reported by Schuster et al. (2012). The deletion status was further supported in this work by diagnostic PCR of the *pkac1* locus and by RT-qPCR, which detected *pkac1* transcripts in QM9414 but not in Δ*pkac1* under the tested culture conditions (Online Resource 16). These strains were grown in liquid Mandels medium containing either 2% (w/v) D-glucose or 1% (w/v) sugarcane bagasse (de Paula et al. 2018) as the sole carbon source. The sugarcane bagasse was kindly donated by Nardini Agroindustrial Ltd., Vista Alegre do Alto, São Paulo, Brazil, and steam-exploded sugarcane bagasse was prepared as previously reported (de Souza et al. 2011). Raw sugarcane bagasse was first treated with steam at 14 kg/cm², followed by extensive washing with distilled water until reducing sugars were undetectable by the 3,5-dinitrosalicylic acid (DNS) assay (Miller 1959). The treated material was then dried at 40 °C for several days.

For phenotypic assays, cultures were inoculated with a spore suspension containing approximately 10^6^ conidia mL^− 1^ and incubated at 30 °C with shaking (200 rpm). Growth was monitored by measuring biomass dry weight and by observing colony morphology on agar plates. Phenotypic and enzymatic assays were performed using three biological replicates per strain unless otherwise indicated. Culture supernatants were collected after 24 h of growth for enzymatic activity assays and protein analyses.

### Enzyme activity assays

The enzymatic activities were measured using the culture supernatants of QM9414 and Δ*pkac1* grown in the presence of sugarcane bagasse. CMCase activity was determined in 96-well PCR microplates, adapting the method originally described by Xiao et al. (2005). The enzymatic reaction was initiated by adding 30 µL of the enzyme solution to 30 µL of 1% carboxymethylcellulose (CMC), which had been prepared in sodium acetate buffer (pH 4.8). The mixture was incubated for 30 min at 50 °C. Subsequent colorimetric detection involved the addition of 60 µL of 3,5-dinitrosalicylic acid (DNS) reagent, followed by heating at 95 °C for 5 min to ensure full color development. Finally, the samples were transferred to a flat-bottomed microplate and the absorbance was measured at 540 nm.

The Filter Paper Activity (FPase) was determined using a small strip of Whatman No. 1 filter paper (50 mg), 30 µL of 100 mM sodium acetate buffer (pH 5.0), and 30 µL of culture supernatant. The reaction was incubated at 50 °C for 30 min. Reducing sugars were quantified by adding 60 µL of DNS reagent followed by heating at 95 °C for 5 min. The absorbance was measured at 540 nm. For β-glucosidase activity, *p*-nitrophenyl-β-D-glucopyranoside (pNPGluc) at a concentration of 5 mM (Sigma-Aldrich, St. Louis, MO, USA) was used. The reaction mixture consisted of 10 µL of the enzyme solution and 40 µL of the pNPGluc substrate solution, prepared in 50 mM sodium acetate buffer, pH 5.5. After incubating the mixtures for 15 min at 50 °C, the enzymatic reaction was terminated by adding 100 µL of 1 M Na_2_CO_3_. For β-xylosidase activity, 10 µL of enzyme solution was incubated with 40 µL of 5 mM *p*-nitrophenyl-β-D-xylopyranoside (pNP-Xyl) in 50 mM sodium acetate buffer (pH 4.8) for 15 min at 50 °C, followed by the addition of 100 µL of 1 M Na_2_CO_3_.

The cellobiohydrolase activity was measured using 5 mM *p*-nitrophenyl-β-D-cellobioside (pNPC) in 50 mM sodium citrate buffer (pH 5.0). The reaction mixture (10 µL supernatant, 40 µL substrate, 50 µL buffer) was incubated at 50 °C for 3.5 h and stopped with 100 µL of 1 M Na₂CO₃. The amount of p-nitrophenol released was subsequently quantified spectrophotometrically by measuring the absorbance at 405 nm.

Finally, xylanase activity was determined by mixing 25 µL of enzyme solution with 50 µL of birchwood xylan (1.0 mg mL^−1^) in 100 mM sodium acetate buffer (pH 5.0) for 30 min at 50 °C. Following the enzymatic incubation, the reaction was stopped, and color development was initiated by adding 75 µL of the DNS reagent. The mixture was subsequently heated at 95 °C for 5 min to allow for the complete chromogenic reaction. The concentration of released reducing sugars was quantified by measuring the absorbance at 540 nm. In CMCase, FPase, β-glucosidase, β-xylosidase and xylanase assays, one enzyme unit (U) was formally defined as the amount of enzyme capable of releasing 1 µmol of reducing sugar per minute (Dos Santos Castro et al. 2014b). For cellobiohydrolase activity, one unit (U) was defined as the amount of enzyme that catalyzes the release of 1 µmol of p-nitrophenol per minute under the specified assay conditions.

### RNA sequencing and transcriptomic analysis

Mycelia were harvested from cultures after 24 h, a time point at which biomass accumulation was comparable between strains, for transcriptomic profiling. RNA-seq was performed using three biological replicates per strain and carbon source. Total RNA was extracted using TRIzol^®^ reagent and purified with silica column kits. cDNA libraries were prepared (poly-A mRNA enrichment) and sequenced on an Illumina platform to generate 150 bp paired-end reads. Quality filtering and adapter trimming of raw reads were performed with standard tools (Chen et al. 2021). High-quality reads were mapped to the *T. reesei* reference genome (version 2.0) using HISAT2 (Kim et al. 2019), and gene expression was summarized as raw read counts per gene. For visualization purposes, normalized abundance values such as fragments per kilobase per million mapped reads (FPKM) were computed. Differentially expressed genes (DEGs) between Δ*pkac1* and QM9414 were identified from raw counts using DESeq2 (Love et al. 2014), with an adjusted *p* value (FDR) < 0.05 as the significance threshold. Genes with significant expression changes were further analyzed for functional enrichment. We performed gene set enrichment analysis on DEGs using FungiFun3 (Garcia Lopez et al. 2024), focusing on Gene Ontology (GO) biological process terms and KEGG pathways associated with metabolism and signaling (Online Resource 6, Online Resource 7, Online Resource 8). The RNA-seq data have been deposited in the NCBI Sequence Read Archive (SRA) under BioProject accession PRJNA1398409.

### Protein extraction and quantitative proteomics

For proteomic analysis, cultures were harvested after 24 h growth in glucose and sugarcane bagasse media using three biological replicates per strain and condition. For total proteome profiling, mycelial pellets were ground in liquid nitrogen and intracellular proteins were extracted in lysis buffer (50 mM Tris-HCl pH 7.5, 8 M urea) supplemented with phosphatase inhibitors (10 mM NaF, 1 mM Na₃VO₄) and a commercial protease inhibitor cocktail (Sigma-Aldrich, St. Louis, MO, USA) to prevent protein degradation and dephosphorylation. In addition, culture supernatants (containing secreted proteins) were collected for secretome analysis. Protein samples were reduced, alkylated and digested with trypsin (Promega) using a standard filter-aided sample preparation protocol. The resulting peptides were desalted and analyzed by high-resolution liquid chromatography-tandem mass spectrometry (LC-MS/MS). An Orbitrap Exploris 240 mass spectrometer (Thermo Scientific) was used for peptide detection, with a nanoLC system for separation. MS/MS spectra were searched against the *T. reesei* protein database using MaxQuant software (Tyanova et al. 2015) for peptide identification and label-free quantification. Differentially abundant proteins between the strains were determined using a t test with permutation-based FDR < 0.05 (as implemented in Perseus; Tyanova et al. 2016). Proteins that were significantly different in abundance between Δ*pkac1* and QM9414 were defined as differentially abundant proteins (DAPs). Functional enrichment analysis of DAPs was performed using FungiFun3 and GO term analysis to identify affected biological processes. The mass spectrometry proteomics data have been deposited to the ProteomeXchange Consortium via the PRIDE partner repository with the dataset identifier PXD072673.

### Phosphoproteomic analysis

To investigate global phosphorylation changes, we performed phosphopeptide enrichment from the tryptic digests using TiO₂ affinity chromatography, using three biological replicates per strain and condition. Enriched phosphopeptides were analyzed on the Orbitrap mass spectrometer in parallel to the total proteome. Phosphosite identification and localization confidence were determined with MaxQuant, employing its built-in phospho (STY) modification search. Quantification of phosphopeptides was done label-free by intensity-based normalization. We compared phosphorylation levels between QM9414 and Δ*pkac1* in both glucose and sugarcane bagasse conditions. Only sites with confident localization (localization probability > 0.75) were considered (Olsen et al. 2006). Differentially abundant phosphorylation sites were identified using two-sample t tests with permutation-based FDR correction in Perseus, applying FDR < 0.05 and |log₂FC| ≥ 1 as significance thresholds. Only phosphosites with localization probability > 0.75 were retained for downstream interpretation. A Venn diagram was constructed to visualize unique and overlapping phosphosites detected in the parental strain but not detected in Δ*pkac1* under each condition (and vice versa) (Fig. 6). Over-representation analysis of GO terms was applied to proteins harboring phosphosites associated with *pkac1* deletion to determine which cellular processes were enriched. Additionally, we scanned the sequences surrounding each phosphosite for the PKA consensus motif (R-R/K-x-S/T). The prevalence of this motif among hypophosphorylated sites in Δ*pkac1* was evaluated by motif enrichment analysis, to infer candidate PKAc1 substrates.

### Molecular docking of candidate PKAc1 substrate peptides

To assess whether certain phosphorylation site motifs correspond to candidate substrates of PKAc1, we conducted in silico peptide docking simulations. We selected several candidate proteins that showed significantly reduced phosphorylation in Δ*pkac1* and contained the PKA consensus sequence. For each candidate, a 9-amino acid peptide encompassing the phosphosite was modeled. A high-confidence 3D structural model of *T. reesei* PKAc1 (catalytic subunit) was retrieved from the AlphaFold database (Jumper et al. 2021). Peptide–protein docking was performed using the HPEPDOCK web server (Zhou et al. 2018), which employs a hierarchical algorithm for blind peptide docking. A PKAr1-derived peptide was used as a reference control for docking. Each candidate peptide was docked into the PKAc1 active site and the top-ranked binding poses were analyzed. Docking scores for the best pose of each peptide were compared to the control peptide’s score (ΔScore = Score^peptide^– Score^PKAr1^) (Online Resource 14). Peptides with a ΔScore close to the PKAr1 peptide were treated as higher-priority candidates for follow-up validation (Online Resource 15; Fig. 8). This approach allowed us to prioritize candidate substrates consistent with PKAc1-dependent phosphorylation for future experimental validation (Fig. 8).

## Results

### Δ*pkac1* exhibits altered growth phenotypes and reduced extracellular enzyme activities on sugarcane bagasse

**Fig. 1:**
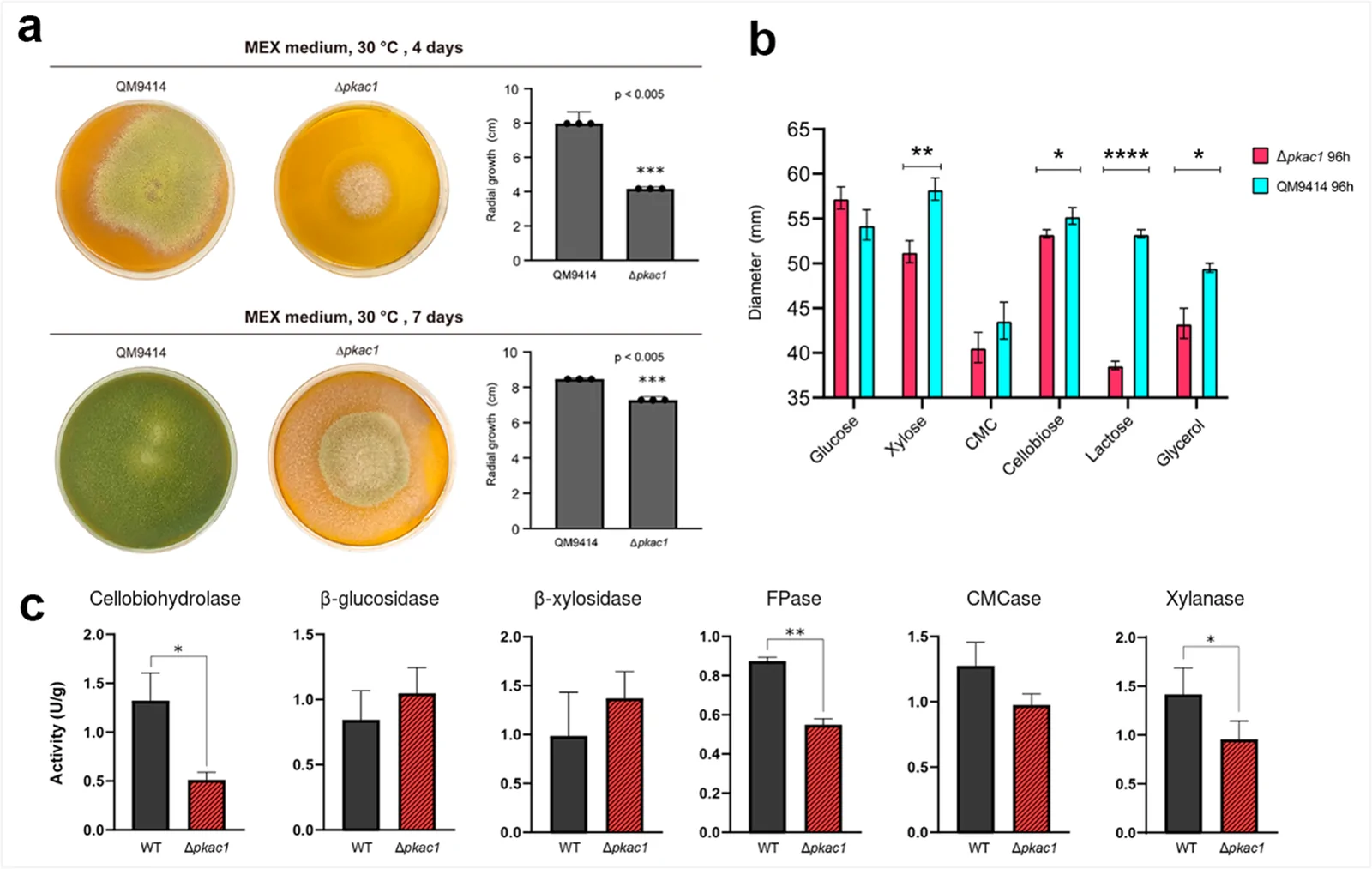
Phenotypic characterization and growth of QM9414 vs. Δ*pkac1*. (**a**) Colony morphology and radial expansion on rich medium (MEX) at 30 °C after 4 days and 7 days; bar plots on the right quantify colony radial growth diameter (mean ± SD, *n* = 3). (**b**) Radial growth diameters at 96 h across carbon sources (glucose, xylose, CMC, cellobiose, lactose and glycerol) for QM9414 and Δ*pkac1*. (**c**) Secreted lignocellulolytic activities after 24 h on 1% sugarcane bagasse (cellobiohydrolase, FPase, CMCase, β-glucosidase, β-xylosidase and xylanase), reported per culture volume (U/mL) and normalized to dry biomass (U/g dry biomass); mean ± SD, *n* = 3

Deletion of the PKA catalytic subunit was associated with altered growth, colony morphology, and pigmentation in *T. reesei*. In the phenotypic characterization across carbon sources, the Δ*pkac1* strain showed slightly reduced radial growth and formed smaller, more compact colonies than the parental strain. In addition, the mutant strain showed loss of the characteristic green pigmentation observed in QM9414 (Online Resource 2). In liquid medium with 1% of glycerol, QM9414 also displayed a higher specific growth rate than Δ*pkac1* (Online Resource 1). These differences were also reflected in the plate-based phenotype, with altered colony morphology (Fig. 1a) and reduced radial growth of Δ*pkac1* when xylose, cellobiose, lactose and glycerol were used as sole carbon sources while no statistical difference was observed on glucose and CMC assays (Fig. 1b).

Consistent with altered growth on biomass, the Δ*pkac1* strain showed an altered extracellular enzyme profile. While the mutant retained the ability to produce accessory enzymes such as β-glucosidase and β-xylosidase at levels comparable to the parental strain (QM9414), the extracellular activities of two depolymerizing enzymes, measured per culture volume, were significantly reduced. Interestingly, cellobiohydrolase and FPase activities showed reductions of approximately 61% (*p* = 0.009) and 36% (*p* = 0.0003) respectively. Similarly, xylanase activity was also reduced in Δ*pkac1* relative to QM9414, whereas CMCase, β-glucosidase, and β-xylosidase were not significantly different between strains. Moreover, because dry biomass at 24 h was comparable between strains (QM9414: 0.051 ± 0.004 g/L; Δ*pkac1*: 0.050 ± 0.004 g/L; *p* = 0.67), these reductions in culture-volume-normalized activity also correspond to reduced activity per unit biomass (Fig. 1c).

Despite these reductions, the enzyme activities on sugarcane bagasse were higher than those in glucose-grown controls (< 0.1 U/g). These results indicate reduced extracellular cellobiohydrolase and FPase activities in Δ*pkac1* under sugarcane bagasse conditions. Given that the two strains displayed divergent growth rates at 10, 16, 20, and 34 h, their physiological states likely capture distinct developmental phases. Such temporal offsets, rather than absolute changes in regulatory capacity, could account for the observed differences (Online Resource 1). To mitigate this variation, all enzymatic and omics measurements were systematically performed at a single standardized time-point (24 h).

### Transcriptomic analysis reveals altered CAZyme-related expression patterns in Δ*pkac1*

To investigate the molecular basis of the reduced enzyme activity phenotype, we compared the transcriptomes of Δ*pkac1* and QM9414 on glucose and sugarcane bagasse. Principal component analysis (PCA) showed tight clustering among biological replicates and clear separation among the four experimental groups, underscoring the reproducibility of the transcriptomic dataset (Online Resource 3). The first principal component (PC1) accounted for 76% of the total variance, primarily resolving the samples by carbon source (glucose vs. sugarcane bagasse), whereas PC2 (12% variance) effectively distinguished the parental QM9414 from the ∆*pkac1* mutant strain under both conditions. Thus, this analysis confirmed that biological replicates grouped robustly, with carbon source and genotype representing the primary and secondary drivers of global transcriptional variance, respectively. Consistent with this distinct clustering, differential expression analysis revealed that *pkac1* deletion triggers condition-dependent reprogramming of the *T. reesei* transcriptome (Online Resource 4, 5).

Differential expression analysis confirmed that *pkac1* deletion triggers robust, widespread transcriptional changes under both conditions, as evidenced by the high density of statistically significant transcripts in both glucose and sugarcane bagasse profiles (Online Resource 4a, b). Moreover, shifting the carbon source from glucose to sugarcane bagasse prompted transcriptomic changes in both the parental and mutant strains (Online Resource 4c, d). Among the alterations promoted by sugarcane bagasse, a core set of 514 annotated genes was shared between the parental and mutant strains and functional enrichment analysis mapped these genes predominantly to primary metabolic pathways, including amino acid metabolism, energy production, and oxidative phosphorylation (Online Resource 6).

**Fig. 2:**
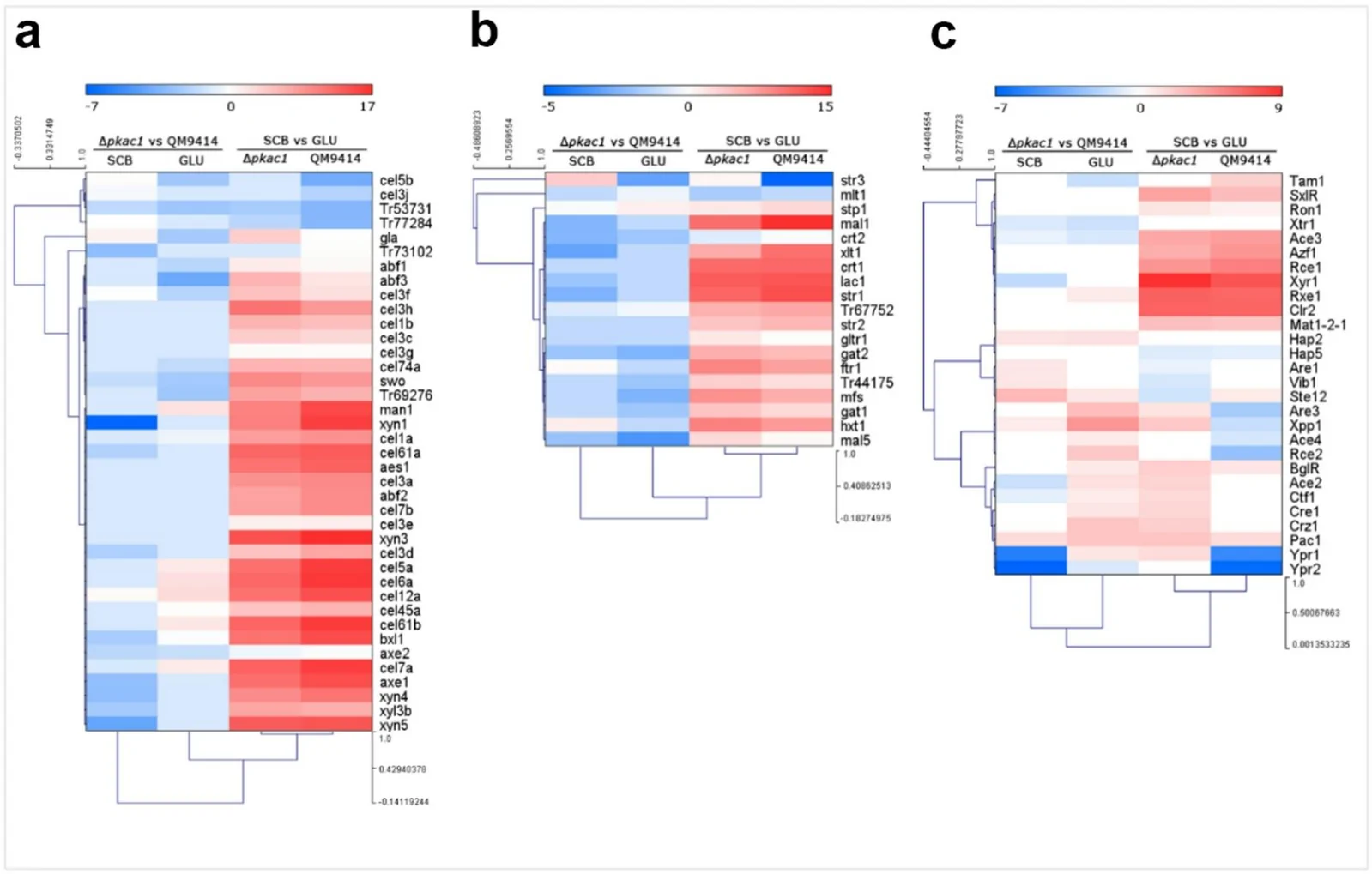
Transcriptomic profiling of CAZymes, transporters, and transcription factors**.** Heatmaps display the relative expression of (**a**) CAZymes, (**b**) nutrient transporters and (**c**) transcription factors in QM9414 and Δ*pkac1* cultivated on glucose or sugarcane bagasse. For each heatmap, columns represent (from top to bottom): Δ*pkac1 versus* QM9414 on sugarcane bagasse (SBC), Δ*pkac1 versus* QM9414 on glucose (GLU), sugarcane bagasse *versus* glucose in Δ*pkac1*, and sugarcane bagasse *versus* glucose in QM9414. Hierarchical clustering highlights sugarcane bagasse-responsive gene programs that show higher expression in the parental strain and reduced amplitude in Δ*pkac1* under these conditions

In sugarcane bagasse, many genes showed significant expression changes in Δ*pkac1* relative to QM9414 (Online Resource 5). Many CAZyme genes normally with increased expression on lignocellulosic substrate showed lower expression levels in Δ*pkac1* relative to QM9414 under sugarcane bagasse conditions (Fig. 2). This included hemicellulases such as *xyn1* (log₂FC = − 1.28, FDR ≤ 0.05) (Fig. 2a), as well as the transcription factor *Ace3* (log₂FC = − 1.22) (Fig. 2c). Although major cellulase genes (*cel7a*/*cbh1* and *cel6a*/*cbh2*) showed lower transcript levels in Δ*pkac1*, these differences did not reach statistical significance (FDR > 0.05) in the direct strain comparison, consistent with attenuated but not abolished induction of lignocellulolytic programs in Δ*pkac1* under the sampled conditions. Heatmap clustering of transcript abundances showed strong carbon-source-dependent induction in QM9414 and a reduced induction amplitude in Δ*pkac1* for multiple CAZyme transcripts (Fig. 2a).

Consistently, several key transcriptional activators governing biomass degradation were repressed in the mutant. For instance, *ace3* transcript levels were significantly diminished in Δ*pkac1* relative to QM9414 (log₂FC = − 1.22, FDR ≤ 0.05), whereas the master regulator *xyr1* exhibited a marginal numerical decline that did not meet the statistical threshold for differential expression (FDR > 0.05) (Fig. 2c). Conversely, transcripts of the carbon catabolite repressor *cre1* were slightly elevated in Δ*pkac1*, suggesting a compromised capacity to fully relieve carbon catabolite repression. The mutant also displayed a downregulation of the cellodextrin transporter *stp1* (log₂FC = − 1.11, FDR ≤ 0.05) alongside variable responses in *crt1*, while expression of the glucose transporter *hxt1* remained unaltered between strains (Fig. 2b). This disrupted transporter profile correlates with affected sensing of cellulose-derived oligosaccharides, ultimately compounding the attenuated expression observed across a substantial subset of CAZyme-encoding genes (Fig. 2a).

**Fig. 3:**
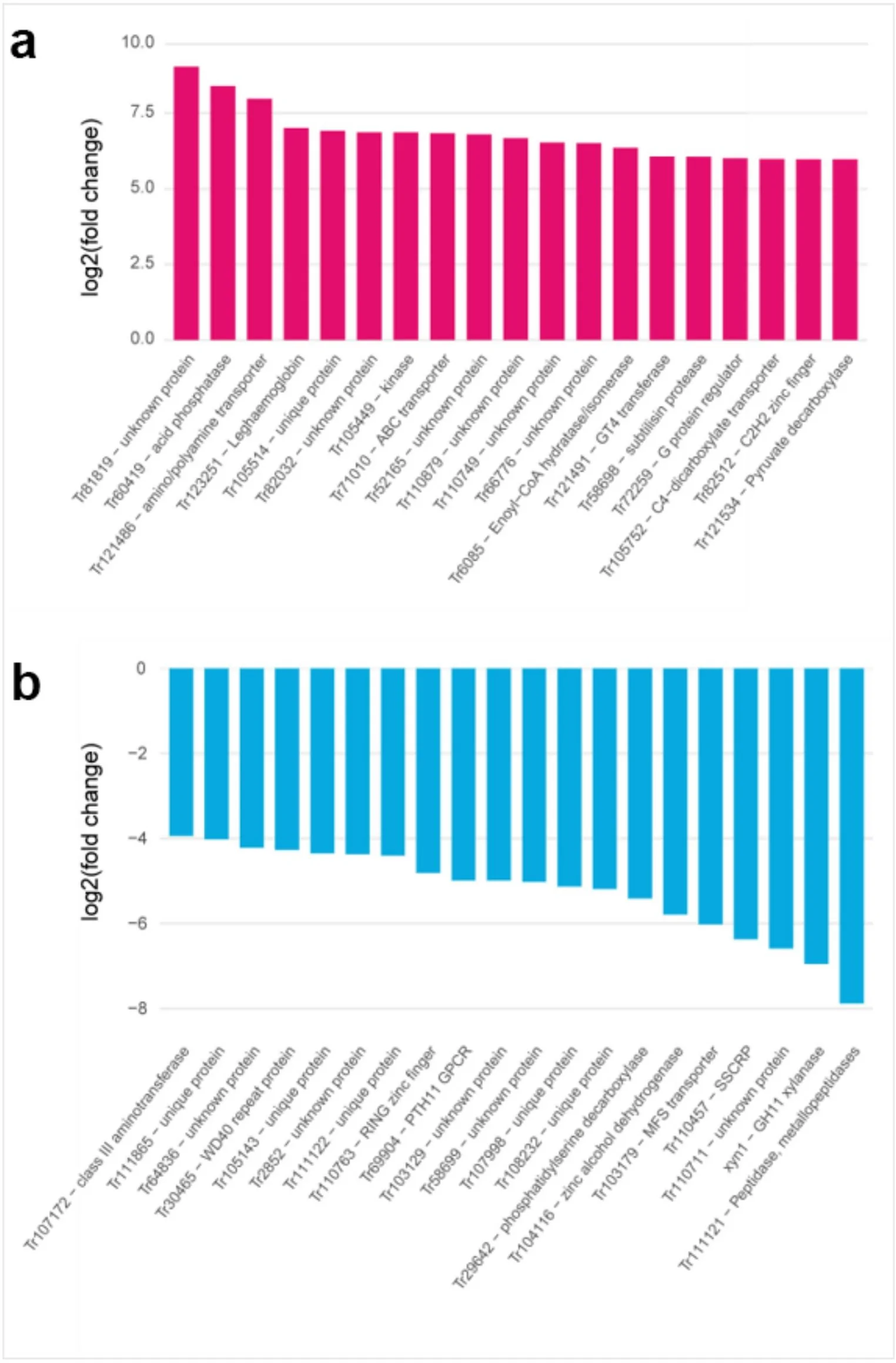
Top differentially expressed genes in Δ***pkac1 versus*** QM9414 on sugarcane bagasse**.** Bar plots showing the top 20 (**a**) up-regulated and (**b**) down-regulated genes (ranked by log₂FC; FDR ≤ 0.05) in the mutant compared to the parental strain under inducing conditions

Notably, the top 20 downregulated genes in Δ*pkac1* relative to the parental strain included signaling-associated and catalytic components, including xyn1, which encodes a GH11 xylanase involved in hemicellulose degradation, alongside a high proportion of uncharacterized or strain-specific proteins (Fig. 3b). This pattern is consistent with PKAc1 contributing to the transcriptional amplitude of part of the hemicellulolytic response at this time point. In contrast, the top upregulated transcripts in the mutant (Fig. 3a) were enriched with genes encoding stress-responsive factors, transporters for alternative nutrients, and hypothetical proteins rather than components of the cellulolytic system. This transcriptional pivot indicates that the ∆*pkac1* strain activates stress-related or survival pathways, potentially triggered by perceived nutrient limitation or altered cell wall dynamics, while simultaneously dampening its response to the complex lignocellulosic substrate. Collectively, these transcriptomic benchmarks demonstrate an attenuated activation of the core lignocellulolytic regulon, aligning with the observed reduction in extracellular enzymatic activities.

### Proteomic profiling reveals reduced abundance of a subset of CAZyme proteins and changes in stress-related pathways

**Fig. 4:**
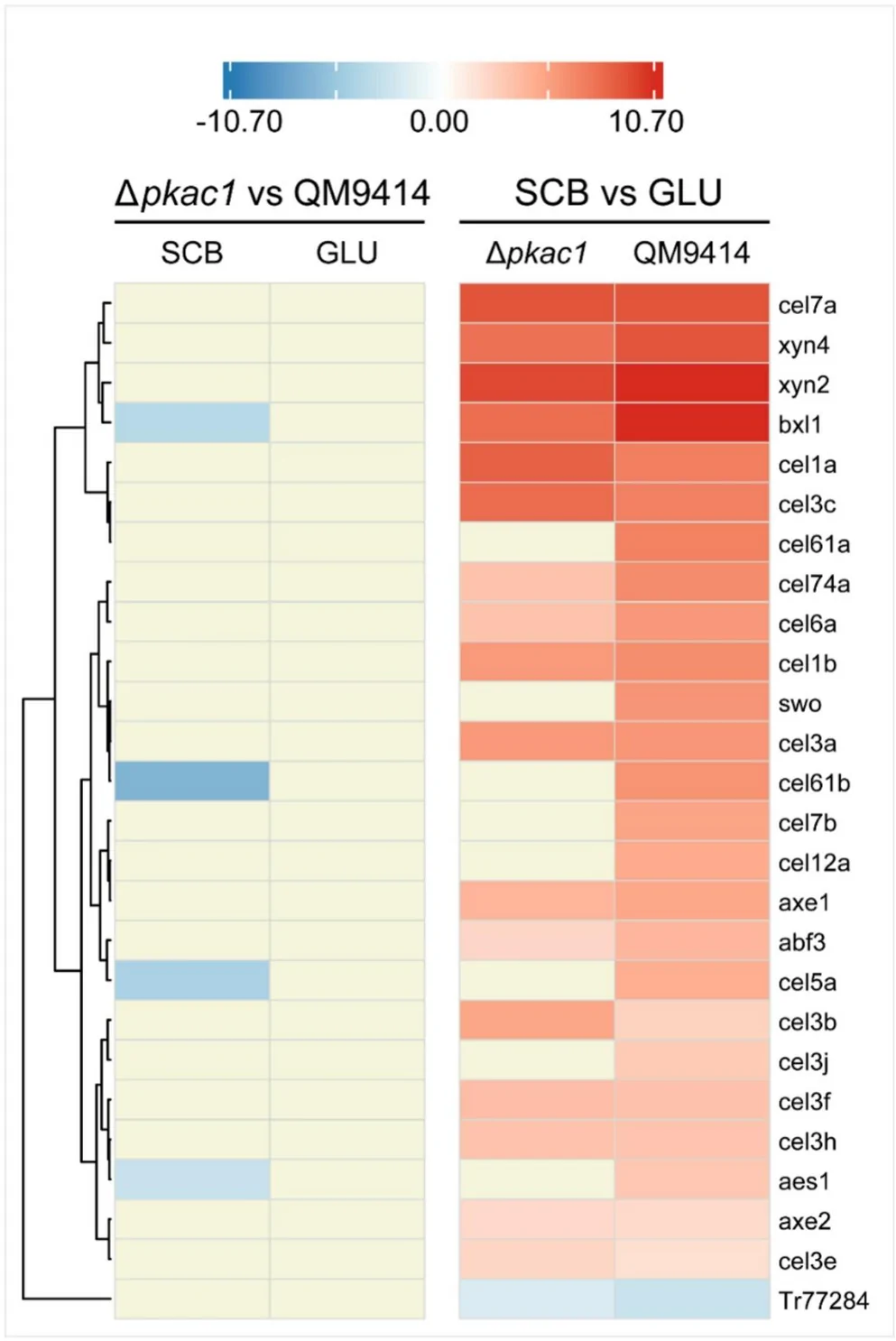
CAZyme protein abundance profiles from proteomics. Heatmap of relative protein abundance (label-free quantification intensity) for selected CAZyme-related proteins across strains and carbon sources. For each columns, the comparison is indicated as follows: Δ*pkac1 versus* QM9414 on sugarcane bagasse (SBC), Δ*pkac1 versus* QM9414 on glucose (GLU), sugarcane bagasse *versus* glucose in Δ*pkac1*, and sugarcane bagasse *versus* glucose in QM9414

We next examined whether the quantitative proteomic profiles reflected the phenotypic and transcriptional differences observed in the mutant strain. To address this, CAZyme-related protein abundance patterns were compared across genotype and carbon-source contrasts (Online Resource 9, 10; Fig. 4). Importantly, Fig. 4 separates direct genotype comparisons from within-strain induction by sugarcane bagasse relative to glucose. In the direct comparison between Δ*pkac1* and QM9414 under sugarcane bagasse, several CAZyme-related proteins showed altered abundance, whereas Cel7A appeared close to neutral in the heatmap. In contrast, the sugarcane bagasse-versus-glucose comparisons showed strong induction of Cel7A and other CAZyme proteins in both strains. Thus, the proteomic data indicate that Δ*pkac1* retains a lignocellulose-responsive CAZyme program, while showing altered abundance patterns for selected CAZyme-related proteins and reduced extracellular activities for specific enzymes.

These data indicate that pkac1 deletion modifies the abundance pattern of selected CAZyme-related proteins without abolishing the induction of major lignocellulolytic enzymes by sugarcane bagasse. The genotype-associated differences observed under sugarcane bagasse should therefore be interpreted together with the within-strain induction patterns and the enzymatic activity data. Overall, the proteomic signatures support a model in which PKAc1 contributes to the magnitude, timing, or downstream processing of selected lignocellulolytic outputs, while Δ*pkac1* retains a measurable response to complex biomass.

**Fig. 5:**
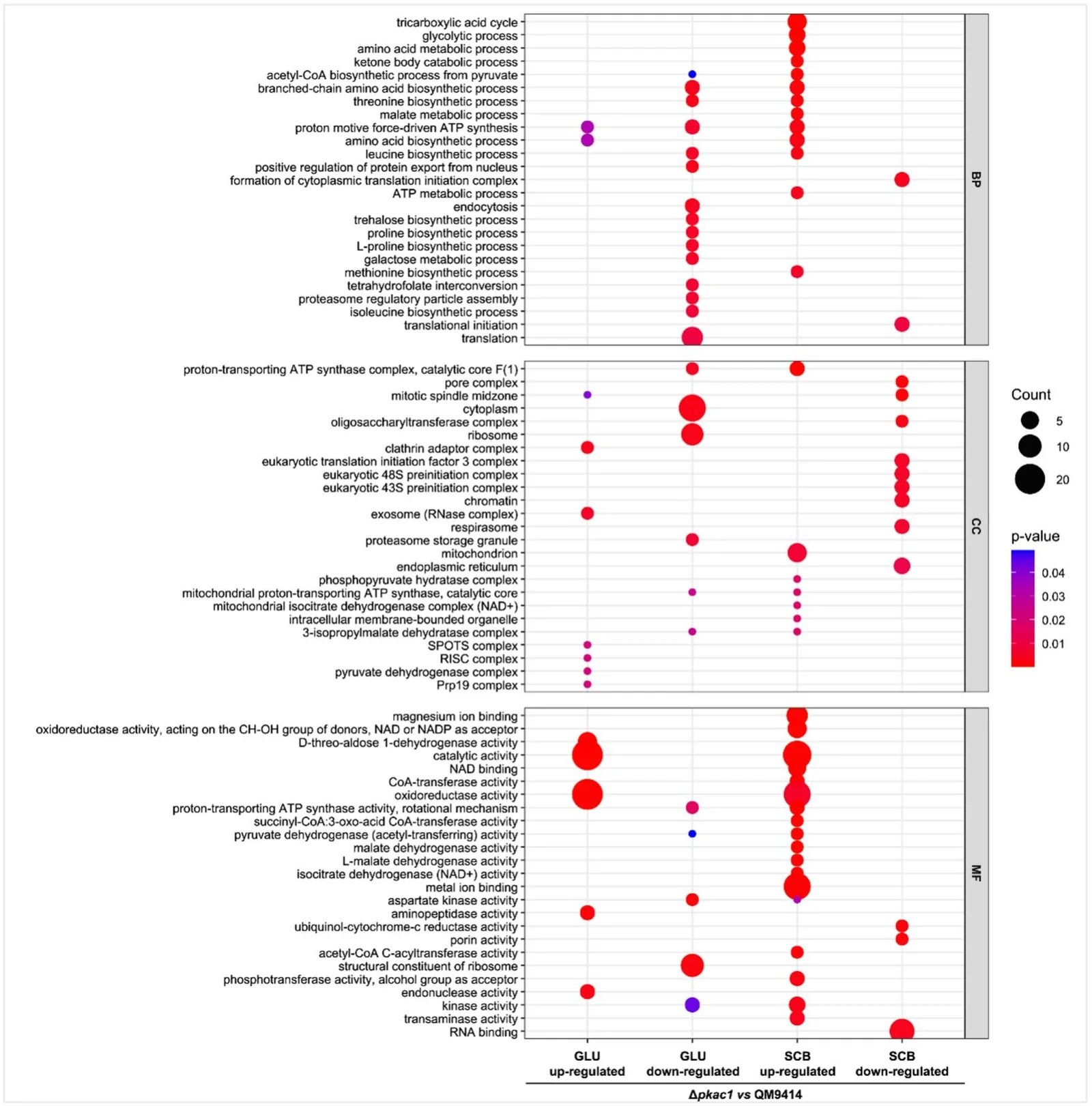
Functional enrichment of differentially abundant proteins. Gene Ontology (GO) significantly enriched (FDR < 0.05) across biological processes (BP), cellular components (CC), and molecular functions (MF). The dot plot contrasts protein with decreased abundance in Δ*pkac1* under sugarcane bagasse challenge (SCB-down-regulated), capturing elements such as translational initiation and pore complexes) against proteins exhibiting increased abundance in the mutant (SCB up-regulated, highlighting core primary and energetic pathways like the tricarboxylic acid cycle, and glycolytic process). Baseline proteomic shifts under repressing conditions are highlighted in the glucose channels (GLU up- and down-regulated). Circle size corresponds to the number of proteins annotated to each term (Count), and the color scale represents statistical significance (p-value)

Interestingly, the proteomic enrichment landscape provided evidence of metabolic reprogramming and compensatory shifts in the mutant strain. GO term enrichment analysis of the proteomic profiles under inductive conditions revealed a structural and metabolic shift in the mutant strain (Fig. 5). Specifically, proteins found at significantly higher levels in ∆*pkac1* under sugarcane bagasse conditions (SCB up-regulated) were predominantly mapped to core primary and energy metabolism pathways (Fig. 5). A striking enrichment was observed for the tricarboxylic acid (TCA) cycle, glycolytic process, and amino acid metabolic pathways within the Biological Process (BP) category. In terms of Cellular Components (CC) and Molecular Functions (MF), these changes were mirrored by the enrichment of mitochondrial structures, such as respirasome and proton-transporting ATP synthase complex, alongside associated enzymatic drivers like oxidoreductase and L-malate dehydrogenase activity (Fig. 5).

In contrast, functional categories highly abundant in the parental QM9414 strain under the same conditions (SCB down-regulated) encompassed elements dedicated to translation initiation, pore complexes, and endoplasmic reticulum dynamics (Fig. 5). This divergent pattern underscores an altered resource allocation profile in the mutant background. These enrichment patterns are consistent with altered resource allocation in the mutant background. While the parental strain showed higher representation of categories related to translation, transport, and biomass-degradation-associated processes, Δ*pkac1* showed increased representation of primary metabolic and energy-associated pathways. These changes may reflect secondary metabolic adaptation to altered carbon-source utilization and growth physiology rather than direct PKAc1 targets.

Beyond the responses to complex lignocellulosic substrate, the proteomic landscape under baseline conditions highlighted that PKAc1-dependent regulation operates constitutively in *T. reesei*, regardless of carbon source complexity (Fig. 5). In a glucose-rich medium, the ∆*pkac1* displayed a significant enrichment of alternative metabolic drivers, including D-threo-aldose 1-dehydrogenase and CoA-transferase activities (GLU up-regulated). Crucially, this baseline shift was accompanied by a robust, carbon-independent overrepresentation of ‘catalytic activity’ and ‘oxidoreductase activity’ domains under both glucose and sugarcane bagasse conditions (GLU up-regulated and SCB up-regulated) (Fig. 5).

Taken together, these data indicate that *pkac1* deletion is associated with basal changes in oxidoreductase-related and metabolic proteins under both glucose and sugarcane bagasse conditions. This pattern suggests that PKAc1 may contribute to basal physiological homeostasis in addition to its association with lignocellulose-responsive outputs. On the other hand, key structural constituents of the ribosome were markedly depleted in the mutant during growth on glucose (GLU down-regulated) (Fig. 5), indicating that translational readiness and ribosome biogenesis are compromised in the mutant prior to any environmental induction.

### Phosphoproteomic profiling reveals PKA-associated signaling networks

**Fig. 6:**
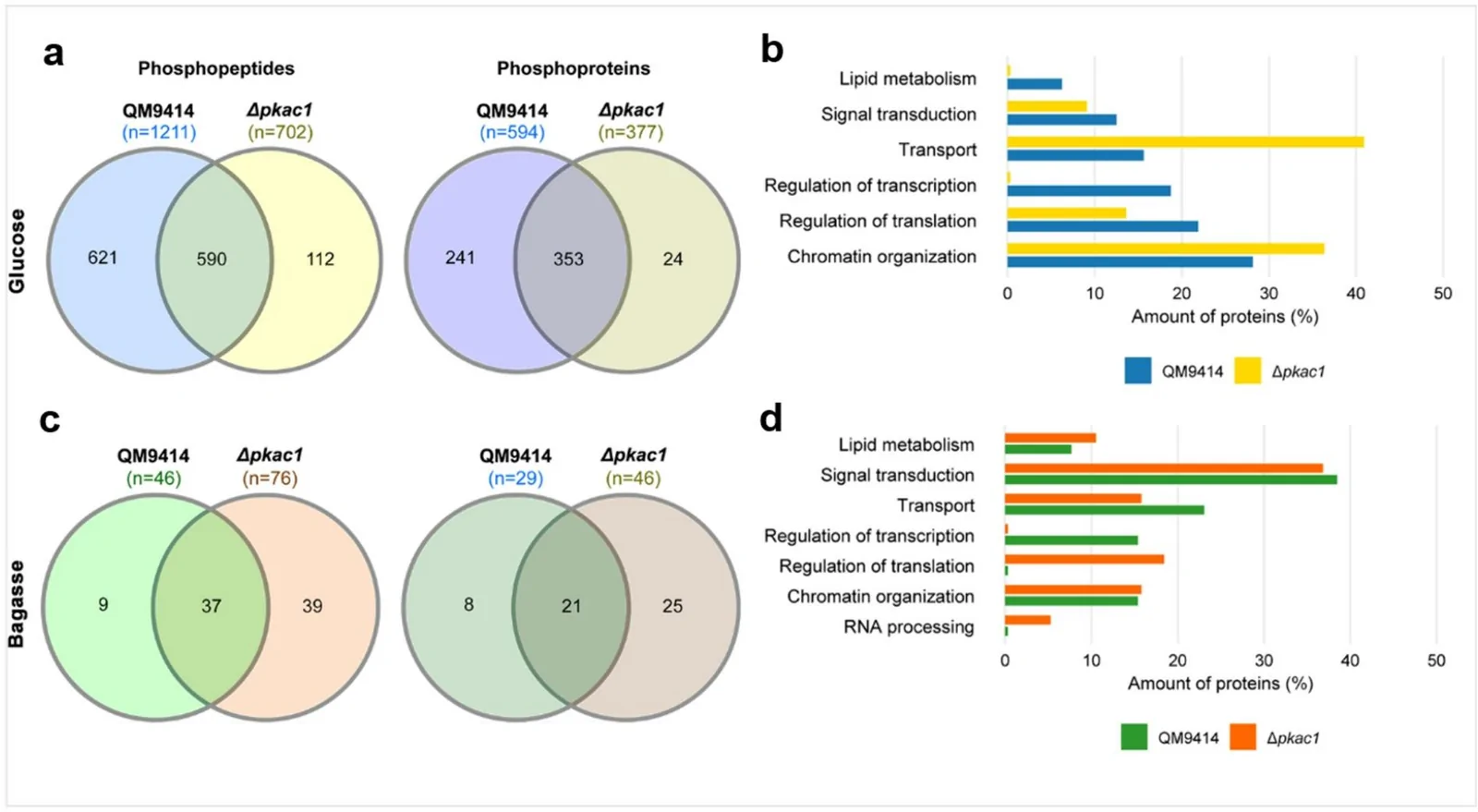
Phosphoproteomics overview. (**a**) Venn diagrams showing unique and shared phosphopeptides and phosphoproteins in QM9414 versus Δ*pkac1* under glucose conditions. The reduced number of phosphosites detected in Δ*pkac1* on glucose is consistent with phosphorylation-state changes associated with *pkac1* deletion. (**b**) GO enrichment for proteins with differentially abundant phosphosites in the parental strain versus Δ*pkac1* in glucose. (**c**) Venn diagrams showing unique and shared phosphopeptides and phosphoproteins in QM9414 versus Δ*pkac1* under sugarcane bagasse conditions. (**d**) GO enrichment for proteins with differentially abundant phosphopeptides and phosphoproteins in the parental strain versus Δ*pkac1* in bagasse. These enrichments indicate condition-dependent changes affecting signaling, transport, and transcriptional/translational regulation

To gain structural insights into phosphorylation-state dynamics associated with the phenotypic remodeling, we mapped the comparative phosphoproteome across both nutritional states (Fig. 6, Online Resource 11). This approach allowed us to identify cellular proteins with altered phosphorylation states in Δ*pkac1*, thereby highlighting candidate PKAc1-associated signaling nodes and potential downstream substrates. The phosphoproteomic data encompassed several thousand phosphorylation sites across the fungal proteome (Online Resource 11).

A broad phosphorylation-state shift emerged when unique and shared phosphosites were compared between strains, with fewer phosphosites detected in Δ*pkac1* than in the parental strain, particularly under glucose conditions (Fig. 6a). In glucose-grown mycelia, the number of unique phosphopeptides detected in the parental strain was higher than in Δ*pkac1* (1,211 versus 702 events; Fig. 6a), suggesting that PKA signaling sustains the basal phosphoproteomic architecture even in the presence of a preferred carbon source. Functional GO enrichment analysis of proteins carrying differentially abundant phosphosites indicated enrichment of categories related to chromatin organization and cellular transport (Fig. 6b). Our analysis also showed that in glucose, only a handful of sites were hyperphosphorylated. Notably, among the hypophosphorylated sites, the consensus motif for PKA (R-R/K-x-S/T) was enriched. Interestingly, many sites matching this motif showed markedly reduced phosphorylation in the mutant (Online Resource 12), suggesting they are candidate PKAc1-associated phosphosites for follow-up validation. Several such sites mapped to proteins associated with cellular regulation and metabolism, consistent with the GO enrichments shown in Fig. 6b and Online Resource 11. The broad decrease in phosphorylation in Δ*pkac1* (Fig. 6a) is consistent with PKAc1 contributing to the phosphorylation-state landscape of selected cellular processes.

Whereas the phosphoproteomic differences were more pronounced under glucose conditions (Fig. 6a, b), the phosphorylation-state profile became more nuanced under sugarcane bagasse conditions (Fig. 6c, d; Online Resource 11). Many sites still showed reduced phosphorylation in the mutant, consistent with PKAc1-associated phosphorylation changes, whereas a subset of phosphosites showed increased phosphorylation in Δ*pkac1* relative to QM9414. Finally, GO term analysis of proteins containing phosphosites associated with *pkac1* deletion revealed enrichment in functions related to signal transduction, transcriptional regulation, and transport (Fig. 6). These categories are consistent with processes potentially influenced by PKAc1-associated signaling during carbon source transition.

Crucially, the phosphoproteomic survey pinpointed specific regulatory proteins whose phosphorylation status was altered in Δ*pkac1*. Among transcription factors, VIB1 (a Zn²⁺-cluster protein implicated in hemicellulase gene induction) had a phosphosite with a PKA-consensus-like sequence that was phosphorylated in QM9414 but strongly reduced in Δ*pkac1* on sugarcane bagasse. Likewise, CLP1 (a putative PHD finger protein that coordinates with Xyr1 during cellulase gene activation) showed reduced phosphorylation in the deletion strain. These changes are consistent with PKAc1-dependent phosphorylation of VIB1 and CLP1, either directly or through intermediate kinases, potentially modulating their activity. Previous studies showed that deletion of *vib1* abolishes cellulase gene induction (Ivanova et al. 2017), and CLP1 is needed for full cellulase expression; therefore, the reduced phosphorylation of these factors in Δ*pkac1* may contribute to the attenuated transcriptional induction observed under these conditions.

The phosphoproteome data also highlighted membrane transporters with altered phosphosites. For example, the STP1 sugar transporter, which transports both glucose and cellobiose, showed a differentially phosphorylated site between strains, consistent with the possibility that PKAc1-associated phosphorylation influences transporter function or trafficking. Additionally, components of the secretory pathway were among the candidate PKAc1-associated phosphoproteins. Notably, the Golgi protein Sect 7 (a guanine nucleotide exchange factor for ARF GTPases) had a site that was hypophosphorylated in Δ*pkac1*. Sect 7 is crucial for vesicle formation and protein secretion; in yeast, cAMP-PKA signaling has been linked to Golgi trafficking, and Sec7 is a key organizer of trans-Golgi function (Richardson et al. 2012; Huang et al. 2014). The reduced phosphorylation of Sec7 in the mutant is consistent with the lower extracellular accumulation of selected enzymes and supports a model in which PKAc1-associated phosphorylation may contribute to secretory pathway function, although direct phosphorylation of Sec 7 by PKAc1 remains to be demonstrated.

**Fig. 7:**
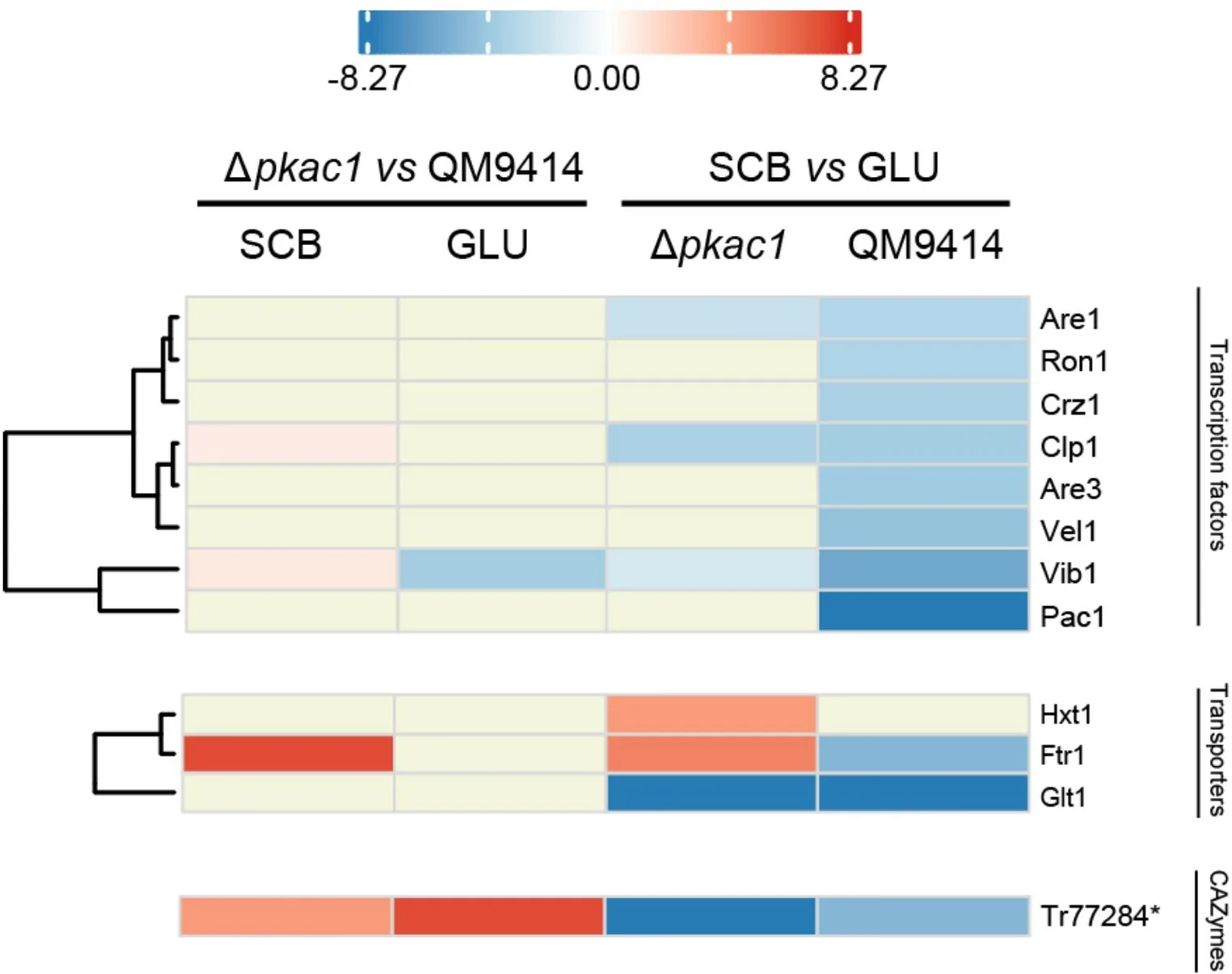
Phosphoprotein heatmaps for selected regulatory and signaling proteins. Heatmaps displaying relative phosphorylation levels (log₂ fold-change Δ*pkac1*/QM9414) for transcription factors, nutrient transporters, and CAZyme. Colors indicate relative phosphorylation changes for each contrast, with blue indicating lower phosphorylation and red indicating higher phosphorylation

Figure 7 summarizes phosphorylation changes in selected CAZyme-related transcription factors, transporters, and signaling or secretion-associated proteins. The heatmap shows distinct condition-dependent phosphorylation patterns rather than a uniform response to pkac1 deletion. For example, Pac1 showed a strong phosphorylation change in the sugarcane bagasse-*versus*-glucose comparison in the parental background, whereas the direct Δ*pkac1 versus* QM9414 comparison under sugarcane bagasse did not support a clear mutant-specific hypophosphorylation signature. Similarly, Are1, Are3, Ron1, VIB1, CLP1, and the selected transporters showed different patterns across genotype and carbon-source contrasts. Therefore, these proteins are best interpreted as candidate regulatory nodes whose phosphorylation states are associated with carbon-source transition and/or *pkac1* deletion. The current data supports a working model in which PKAc1-associated phosphorylation changes may intersect with transcriptional regulation, transporter function, and secretion-associated processes.

### Docking prioritizes candidate PKAc1 substrates among phosphoproteins

**Fig. 8:**
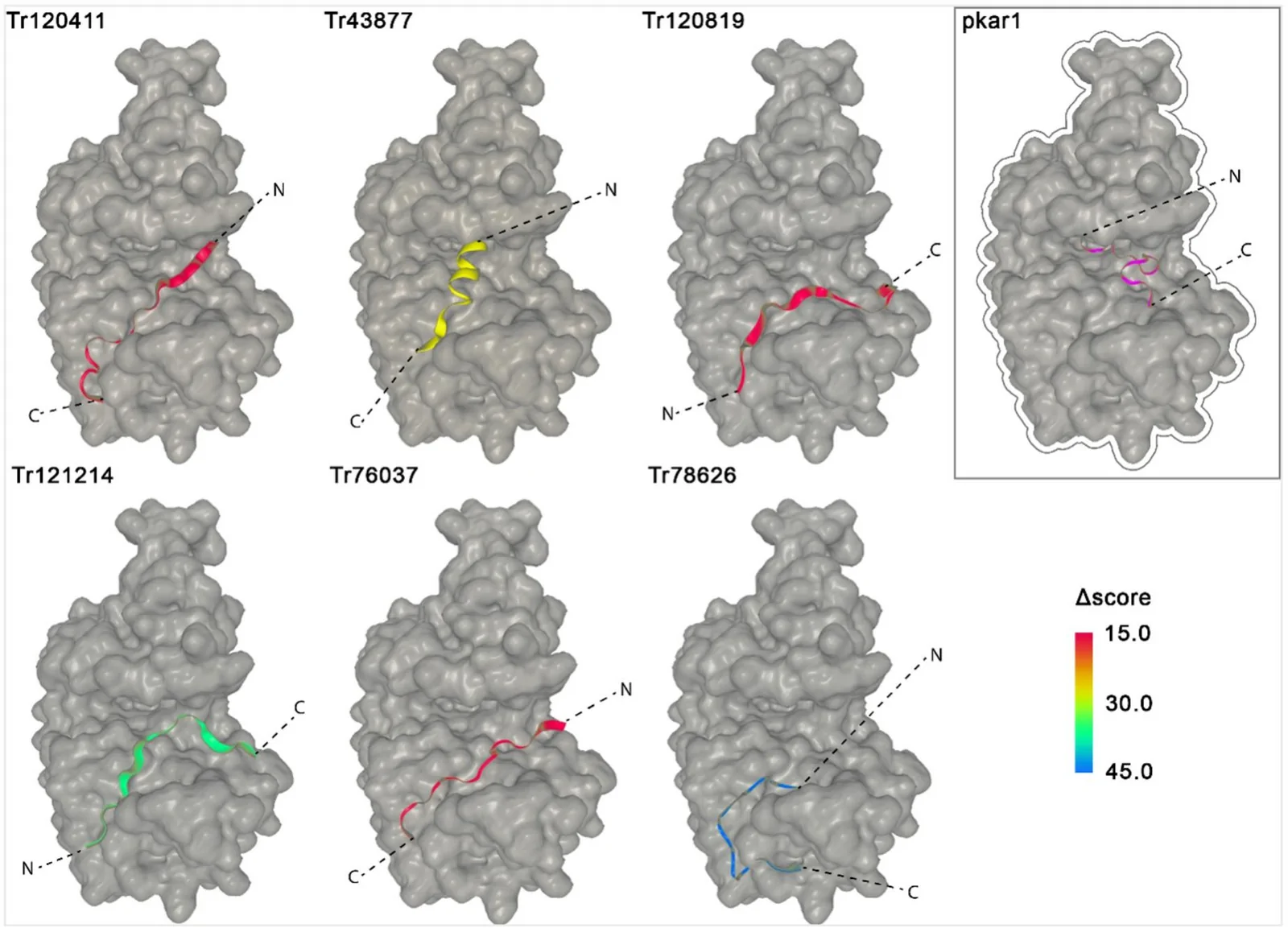
Docking of candidate PKAc1-associated substrate peptides**.** Structural model of the *T. reesei* PKAc1 catalytic cleft (gray surface) docked with high-priority candidate peptides (colored ribbons) and the reference inhibitor peptide (PKAr1, magenta). Candidate peptides are labeled by their protein identifiers (Tr IDs). The color bar represents comparative docking scores (∆Score relative to PKAr1), where warmer tones (red/orange) indicate more favorable predicted docking scores within the catalytic groove, and cooler tones (blue/green) indicate less favorable predicted docking scores. These predicted docking results prioritize candidate molecular nodes for follow-up functional validation

To prioritize altered phosphorylation sites that could represent candidate PKAc1 substrates, we performed in silico docking of candidate peptide motifs into the PKAc1 catalytic cleft (Fig. 8). We focused on candidates that contained the PKA consensus motif (R-R/K-x-S/T) and showed reduced phosphorylation in Δ*pkac1* (Online Resource 12). For each candidate, a 9-residue peptide window encompassing the localized serine/threonine phosphosite was docked into the predicted *T. reesei* PKAc1 structure (Fig. 8; Online Resource 14). A PKAr1-derived peptide was used as a reference control for docking (Fig. 8).

The docking analysis ranked candidate peptides according to their predicted interaction scores relative to the PKAr1-derived reference peptide (Online Resource 14, 15). The top-ranked peptides included candidates derived from Tr76037, Tr120819, Tr120411, and Sect7/Tr43877. Among these, Tr76037 and Tr120819 showed the closest ΔScore values relative to the PKAr1 control, whereas Tr120411 was the highest-ranking candidate detected under sugarcane bagasse conditions (Fig. 8; Online Resource 14, 15). The Tr120411-derived peptide (HRRKSSTHH) contains the PKA consensus motif and showed a favorable predicted docking pose within the PKAc1 catalytic cleft. These results prioritize candidates for downstream validation.

Although the downstream functional roles of several candidate proteins remain uncharacterized, the convergence of PKA-consensus motif presence (Online Resource 14), reduced phosphorylation in Δpkac1 (Online Resource 12), and favorable predicted docking behavior (Fig. 8) provides a prioritized shortlist of candidate PKAc1-associated substrates for follow-up validation. However, docking scores, consensus motifs, and phosphorylation changes in Δ*pkac1* do not establish direct kinase-substrate relationships; biochemical assays and phosphosite mutagenesis will be required to validate direct phosphorylation and functional relevance.

### Multi-omics integration reveals transcript-protein discordance in Δ*pkac1*

**Fig. 9:**
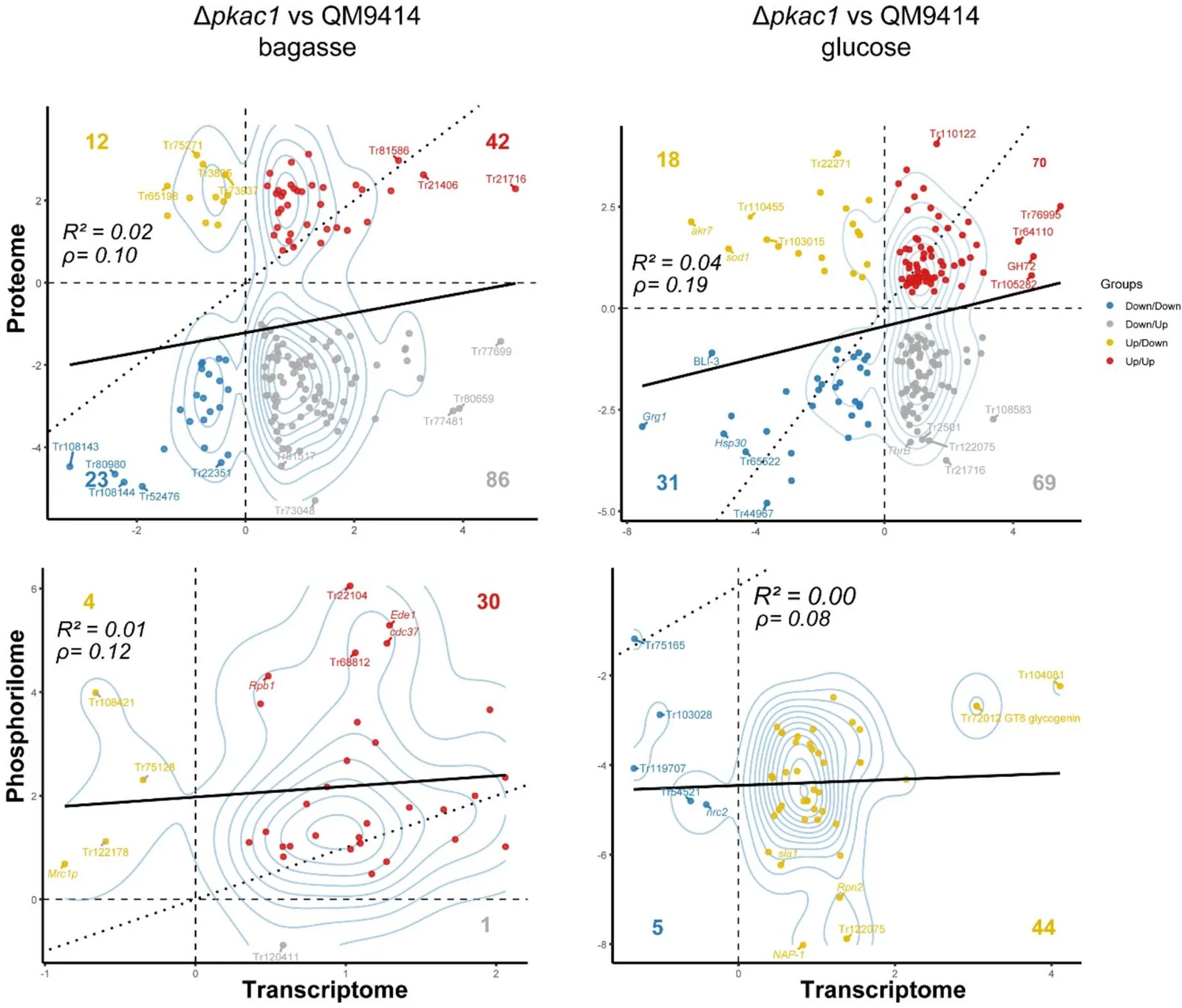
Transcript-protein and transcript-phosphosite coordination profiles in Δ*pkac1*. Multi-omic scatterplots mapping log_2_ fold changes (∆*pkac1*/QM9414) with the transcriptomic landscape plotted on the x-axis. Top row: y-axis displays total protein abundance changes (proteome). Bottom row: y-axis maps localized phosphorylation alterations (phosphoproteome at the site/peptide level). Left column isolates sugarcane bagasse condition and the right column captures glucose repression. Individual points are color-coded based on specific quadrant-membership distribution (Down/Down, Down/Up, Up/Down, and Up/Up, as detailed in the panel legend). Solid lines represent the fitted linear regression models, and kernel-density contours resolve regions of high data concentration. Notably, across both carbon sources and signaling layers, consistently low determination coefficients (R² ≤ 0.04) and data dispersion relative to the theoretical 1:1 parity diagonal line indicate limited proportionality between transcript abundance, protein abundance, and phosphorylation-state changes

By integrating the multi-omic layers, we observed an imperfect correspondence between transcript accumulation and downstream molecular outputs in the ∆*pkac1* mutant strain, indicating transcript-protein and transcript-phosphosite decoupling across both carbon sources (Fig. 9). In the parental strain, the induction of a gene’s transcript generally coincided with an increased abundance of the encoded protein, especially for key components of the secretome. In ∆*pkac1*, however, while multiple cellulase transcripts were still successfully detected under inducing conditions, the corresponding proteins accumulated to markedly lower levels than in the QM9414 background (Fig. 9). Conversely, a distinct subset of genes displayed modest transcript variations but outsized protein-level discrepancies, indicating that transcriptomic abundance alone is insufficient to predict the absolute translation or stability of the enzymatic machinery.

A comprehensive cross-omics comparison of fold changes illustrates the limited correspondence between transcript-level changes and changes in protein or phosphosite abundance (Fig. 9). Across all examined comparisons, low coefficients of determination (R² ≤ 0.04) and shallow regression slopes indicated weak concordance between omics layers. Kernel-density contours and the frequent positioning of points below the 1:1 parity line indicate that, for many matched features, protein-abundance or phosphosite fold changes were more negative or less positive than the corresponding transcript fold changes. Because all measurements were obtained at a single 24 h time point, these differences may reflect temporal offsets together with post-transcriptional and post-translational regulation; the present data do not distinguish among these possibilities.

The quadrant-membership mapping within the total proteome layer demonstrated that the regulatory imprint of the PKAc1 pathway is constitutively operational, exerting continuous baseline control regardless of substrate complexity (Fig. 9). Under glucose conditions (top-right panel), a prominent cohort of 70 targets was robustly and coordinately up-regulated at both the mRNA and total protein levels in the mutant strain (Up/Up quadrant). This constitutive molecular signature significantly outnumbered the 42 Up/Up targets captured during sugarcane bagasse challenge (top-left panel). In contrast, the transcript-phosphosite correlation (bottom row) revealed a much narrower, condition-dependent architecture (Fig. 9). Under sugarcane bagasse cultivation, a discrete group of 30 targets maintained a synchronized Up/Up positioning (bottom-left panel), whereas growth on simple sugars restricted the shared distribution to a minimal cluster of only 5 Down/Down entities (bottom-right panel). Together, these scatterplot profiles support a multilayered model in which transcriptomic reprogramming provides only one component of the Δ*pkac1* response, whereas protein abundance and phosphorylation-state changes contribute additional regulatory layers. The current data indicate that PKAc1-associated effects involve transcriptional, post-transcriptional, post-translational, and secretion-associated processes.

## Discussion

**Fig. 10:**
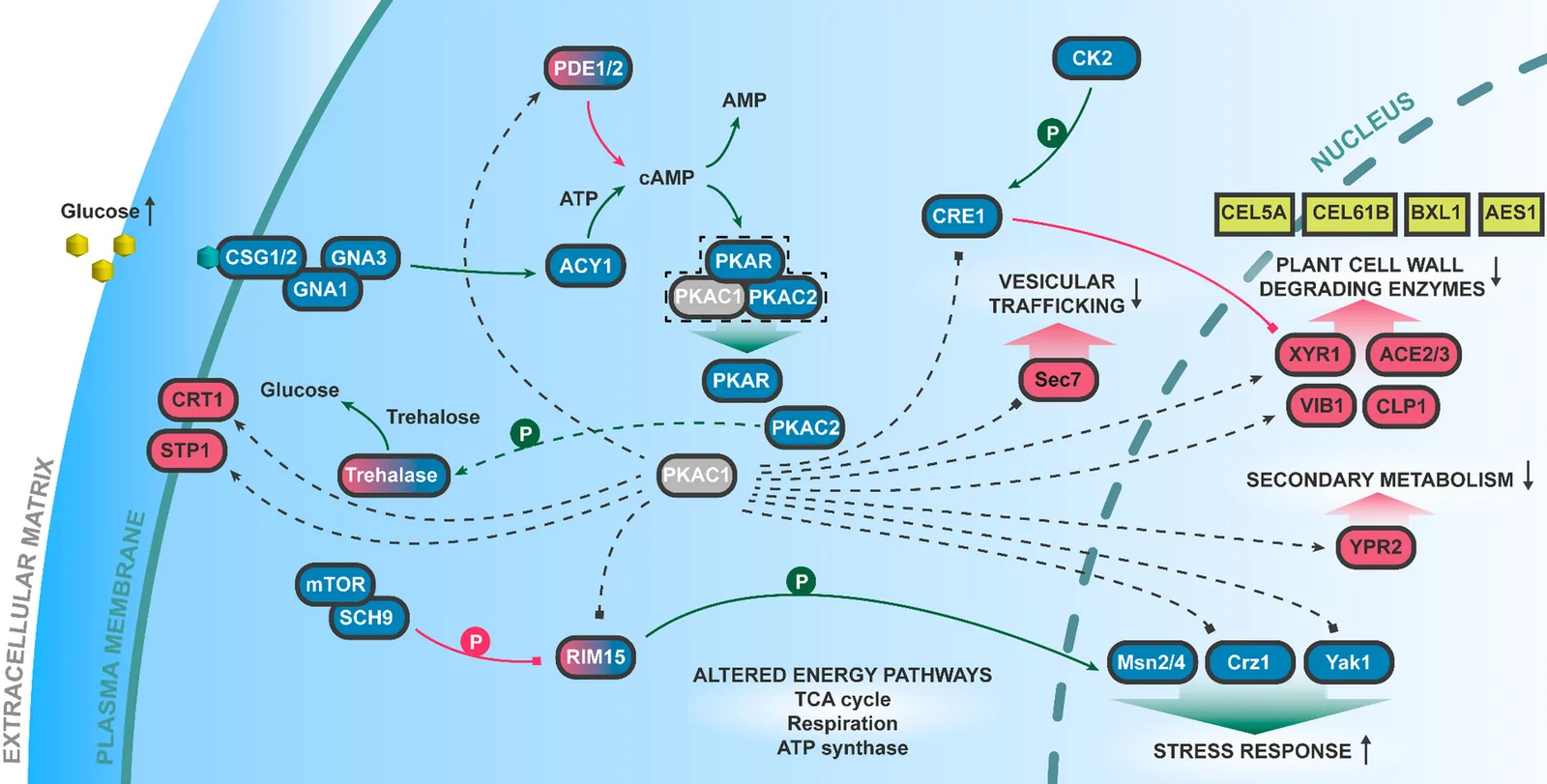
Working model for PKAc1-associated regulation of the lignocellulolytic response**.** In the parental strain, carbon source sensing and cAMP–PKA signaling are proposed to influence the lignocellulolytic response through multiple regulatory layers. PKAc1-associated phosphorylation may contribute to the regulation of inducer transporters such as Crt1 and Stp1, modulation of transcription factors including CRE1, XYR1, ACE2/3, VIB1, and CLP1, and secretion-associated processes involving Sec7 and vesicular trafficking. The model also incorporates broader physiological adaptations associated with altered energy metabolism, including changes in the TCA cycle, respiration, and ATP synthase-related processes, as well as stress response pathways. In Δ*pkac1*, altered phosphorylation across these nodes is consistent with retained but altered sugarcane bagasse responsiveness, modified CAZyme-related output, transcript–protein/phosphosite decoupling, changes in secondary metabolism and stress adaptation, and reduced extracellular activity of selected biomass-degrading enzymes under the sampled conditions. Colors indicate proposed activity states: blue, active; pink, inactive or strongly reduced; and blue/pink, partially active or attenuated

Our integrated multi-omics analysis indicates that *pkac1* deletion is associated with multilayered regulatory remodeling in *T. reesei* under glucose and sugarcane bagasse conditions. By combining transcriptomic, proteomic, and phosphoproteomic datasets, we observed condition-dependent changes in gene expression, protein abundance, and phosphorylation states in Δ*pkac1* relative to the parental strain. Rather than supporting a complete loss of lignocellulolytic induction, the combined data indicate an attenuated and redistributed response affecting selected CAZyme-related outputs, nutrient transporters, stress-associated proteins, metabolic pathways, and candidate phosphorylation targets (Figs. 9 and 10).

The presence of 70 core targets coordinately up-regulated at both the mRNA and protein levels (Up/Up quadrant) during growth on glucose significantly outnumbers the 42 targets captured under sugarcane bagasse induction (Fig. 9). Functional enrichment of proteins harboring altered phosphosites under glucose conditions highlighted categories related to chromatin organization and cellular transport (Fig. 6b), suggesting that PKAc1-associated phosphorylation may influence these processes under baseline conditions. The transcript levels of the second PKA catalytic subunit gene, *pkac2*, remained unaltered in the deletion strain (FDR > 0.05), indicating that the residual response is not driven by a compensatory upregulation of the paralog. However, because all omics measurements were collected at a single time point, some differences may reflect growth-phase offsets, delayed adaptation or altered response amplitude. Notably, dry biomass was comparable between strains at 24 h.

One of the key observations is reduced induction of multiple CAZyme genes in Δ*pkac1* under the sampled inducing condition. This pattern is consistent with PKA acting upstream of, or in parallel with, major transcriptional regulators such as Xyr1 that drive cellulase and hemicellulase gene expression. In the parental strain, the shift from a preferred carbon source to an insoluble substrate like sugarcane bagasse triggers a coordinated transcriptional program involving derepression and activation of inducer-sensing pathways. Our results suggest that PKA contributes to this transition. In Δ*pkac1*, *xyr1* transcript levels were numerically lower than in QM9414 under sugarcane bagasse conditions, although this difference did not reach the DEG significance threshold (FDR > 0.05). Nevertheless, several downstream CAZyme genes showed reduced expression relative to the parental strain, consistent with previous reports linking PKA signaling to cellulase gene regulation (Hinterdobler et al. 2019; Li et al. 2022). Notably, major cellulase genes (*cel7a*, *cel6a*) were still robustly induced in Δ*pkac1*, indicating attenuation rather than complete loss of induction. The reduced expression of *stp1* and variable responses among other transporters such as *crt1* are consistent with altered carbon source sensing in the deletion strain. Consistent with this, the mutant transcriptome revealed misregulation of G-protein coupled receptors (GPCRs), including members of the PTH11 family, which serve as crucial upstream sensors for complex carbohydrates in fungi. We hypothesize that PKAc1 contributes to the sensing of glucose exhaustion and the response to alternative carbon cues. Without PKAc1, the cell may have a reduced capacity to transmit the signal that complex polysaccharide is available, contributing to prolonged carbon catabolite repression and attenuated induction of cellulolytic activators.

At the post-translational level, the phosphoproteomic data shed light on how PKAc1-dependent phosphorylation may modulate critical regulators. We identified two transcription factors, VIB1 and CLP1, whose phosphorylation was reduced in Δ*pkac1*. VIB1 in *T. reesei* is implicated in hemicellulase gene induction and integrates carbon signaling in *N. crassa*. The hypophosphorylation of VIB1 in Δ*pkac1* is therefore consistent with the possibility that PKAc1 supports VIB1 activity, either directly or through intermediate kinases. Ivanova et al. (2017) linked loss of *vib1* expression to impaired cellulase induction and showed that *vib1* deletion reduced cellulase expression in QM9414 and Rut-C30, supporting VIB1 as a plausible node linking phosphorylation-state changes to altered transcriptional output. Likewise, reduced CLP1 phosphorylation may diminish its ability to co-activate cellulase genes with Xyr1 (Wang et al. 2019). These findings suggest that PKAc1 may influence transcription-factor activity through phosphorylation-state changes, but direct kinase-substrate relationships will require targeted validation.

Another insight is that PKAc1-dependent phosphorylation may be linked to secretory pathway capacity and cellular capacity for enzyme output. We observed global hypophosphorylation of proteins involved in translation, energy metabolism and secretion in Δ*pkac1*, particularly under repressing conditions. During induction, *T. reesei* needs to increase protein synthesis and secretion to export large quantities of enzymes, and PKAc1 may contribute to this ramp-up. The hypophosphorylation of Sec7 in Δ*pkac1* is particularly notable because Sec7 is a trans-Golgi network-localized Arf guanine-nucleotide exchange factor. In *Candida albicans*, cAMP–PKA signaling has been linked to Golgi trafficking through PKA-dependent phosphorylation of Gyp1, which also influences Sec7 recruitment (Richardson et al. 2012; Huang et al. 2014). These observations provide precedent for a connection between PKA signaling and Sec7-associated Golgi organization, although they do not establish Sec7 as a direct PKA substrate. Our results are therefore consistent with the possibility that PKAc1-dependent phosphorylation helps support secretory pathway capacity when enzyme production is required. In the absence of PKAc1, Golgi and vesicle trafficking processes may operate less efficiently, which could contribute to lower extracellular accumulation of enzymes. This lower extracellular enzyme accumulation is also accompanied by increased abundance of specific Rab GTPases and SNARE proteins in the mutant, suggesting possible compensatory changes in vesicular routing or membrane fusion mechanisms. However, the relative contributions of secretion, growth rate, and induction kinetics remain to be resolved by future time-course experiments.

Our docking analysis provides a shortlist of candidate PKAc1 substrates for validation. The highest-ranking peptides included one derived from Sec7 and three derived from proteins of unknown function. These results help prioritize candidate substrates with docking behavior consistent with the phosphoproteomic data. In this sense, the docking results support a model in which PKAc1 may act at multiple nodes of the regulatory network, potentially affecting secretion, transcriptional regulation and transporter-associated processes. This broader view is consistent with observations in other filamentous fungi, where PKA influences multiple downstream pathways. Our work extends this framework to an industrial enzyme producer and specifically to its lignocellulolytic response.

It is informative to compare our results with previous studies of PKA signaling in *T. reesei*. Schuster et al. (2012) reported strong effects of PKA pathway components on light-modulated cellulase regulation and growth-related phenotypes, whereas Li et al. (2022) described reduced cellulase activity together with pigmentation and sporulation phenotypes in a pkac1 deletion strain in the QM6a background. The milder plate-growth phenotype and retained lignocellulolytic induction observed here may reflect differences in genetic background, strain history, medium composition, cultivation format, incubation time, carbon source, and growth quantification criteria. Our findings in QM9414 are therefore best interpreted as extending previous work by showing that *pkac1* deletion is associated with multilayered transcriptomic, proteomic, and phosphoproteomic remodeling.

The revised model can be viewed as a conceptual hierarchy of evidence in which the most proximal layer consists of candidate PKAc1-associated phosphosites and peptides containing PKA-like motifs, including candidates prioritized by docking. A second layer includes candidate regulatory and trafficking nodes, such as transcription factors, transporters, and Sec7-associated vesicular trafficking, which may connect phosphorylation-state changes to CAZyme-related outputs. A third layer includes broader metabolic and stress-associated changes, such as TCA cycle, respiration, oxidoreductase, and energy-metabolism signatures, which are most conservatively interpreted as secondary or indirect adaptations. This hierarchy helps distinguish candidate proximal kinase-associated events from downstream physiological remodeling. The combined approach of transcriptomics, proteomics/secretome profiling, phosphoproteomics, motif analysis, and peptide docking within the same *pkac1* mutant framework provides a systems-level view of PKAc1-associated regulatory remodeling and prioritizes candidate regulatory nodes for future validation.

In summary, our study provides a multi-layered view of how PKAc1 deletion is associated with changes across the transcriptome, proteome, and phosphoproteome of *T. reesei*. Figure 10 presents a working model that summarizes these patterns. In this model, PKAc1-associated phosphorylation may help connect carbon-source sensing to transporter behavior, transcription-factor activity and secretory pathway capacity during growth on sugarcane bagasse. In Δ*pkac1*, altered phosphorylation of these nodes is consistent with attenuated CAZyme induction and lower extracellular enzyme accumulation under the sampled conditions. This model is intended as a hypothesis-generating framework and targeted experiments will be needed to determine which links are direct.

These results reinforce why PKA is often described as a pleiotropic regulator in fungi (Kerkaert and Huberman 2023). By prioritizing candidate substrates or PKAc1-associated phosphoproteins such as Sect 7 and VIB1, we provide testable leads for downstream validation and for future strain-engineering efforts. Any engineering application, however, will depend on direct functional tests of the candidate phosphorylation events identified here.

By integrating multiple omics layers, we observed that PKAc1 deletion is associated with changes in cellulase gene expression, phosphorylation states and secretion-associated processes during growth on lignocellulosic substrate in *T. reesei*. These findings expand our understanding of carbon signaling in filamentous fungi while also highlighting the limits of inference from a single multi-omics snapshot. Future studies should resolve the temporal contribution of growth-phase effects, secretion-associated processes, and direct kinase-substrate relationships through time-course experiments, phosphosite mutagenesis, and biochemical validation. Candidate phosphorylation events could be tested using phospho-ablative substitutions, such as Ser/Thr-to-Ala, and phosphomimetic substitutions, such as Ser/Thr-to-Asp or Ser/Thr-to-Glu. Functional follow-up should also include cellular localization assays, targeted secretome profiling, enzyme activity measurements, in vitro kinase assays, and validation of candidate nodes such as Sect 7, selected transporters, and transcriptional regulators.

## Conclusions

PKAc1 contributes to regulatory adaptation of *T. reesei* to lignocellulosic substrate. Across transcriptomic, proteomic, and phosphoproteomic layers, *pkac1* deletion was associated with altered growth-related phenotypes on sugarcane bagasse, reduced extracellular activities of selected lignocellulolytic enzymes per culture volume, and broad phosphorylation-state changes enriched for the PKA consensus motif. These multi-omics patterns support a working model in which PKAc1-associated phosphorylation may contribute to carbon-source sensing, regulatory outputs and secretion-associated processes. The present dataset supports attenuated induction for several CAZyme-related responses but the relative contributions of growth-phase effects, transcriptional regulation, secretion-associated processes and direct kinase-substrate relationships will require time-course and targeted validation.

## Supplementary Information

Below is the link to the electronic supplementary material.

Supplementary File 1 (PDF 2.00 MB)

## Data Availability

No datasets were generated or analysed during the current study.

## Materials availability

The T. reesei strains and non-commercial biological materials described in this study are available from the corresponding author upon reasonable request, subject to institutional policies and applicable material transfer agreements.
