# Supplementary material for: Multi-omics analysis reveals Protein Kinase A-associated regulatory remodeling during adaptation of Trichoderma reesei to lignocellulosic substrate
Source: World J Microbiol Biotechnol. 2026 Sep 8;42(9):523. doi: 10.1007/s11274-026-05252-x (PMC13550043; doi:10.1007/s11274-026-05252-x)

Electronic Supplementary Material

Multi-omics analysis reveals protein kinase A-associated regulatory remodeling during adaptation of *Trichoderma reesei* to lignocellulosic substrate

World Journal of Microbiology and Biotechnology

Hermano Zenaide Neto[1], Wellington Ramos Pedersoli[1], David Batista Maués[1], Lucas Matheus Soares Pereira[1], Iasmin Cartaxo Taveira[1], Thiago de Andrade Simon[4,5], Andrei S Steindorff[2], Vitor Marcel Faça[3], Renato Graciano de Paula[4,5], Roberto N Silva[1,5*]

[1]Department of Biochemistry and Immunology, Molecular Biotechnology Laboratory, Ribeirão Preto Medical School (FMRP), University of São Paulo, Ribeirão Preto 14049-900, SP, Brazil

[2]Joint Genome Institute, Lawrence Berkeley National Laboratory, Berkeley, CA, 94720, USA.

[3]Department of Biochemistry and Immunology, Cancer Proteomics Laboratory, Ribeirão Preto Medical School (FMRP), University of São Paulo, Ribeirão Preto 14049-900, SP, Brazil

[4]Department of Physiological Sciences, Health Sciences Center, Laboratory of Structural and Functional Biochemistry, Federal University of Espírito Santo, Vitória 29047-105, ES, Brazil

[5]National Institute of Science and Technology in Human Pathogenic Fungi, Brazil

***Corresponding author:** rsilva@fmrp.usp.br

Online Resource 1. Growth kinetics of *T. reesei* QM9414 and Δ*pkac1* in liquid medium containing glycerol. Strains QM9414 (blue) and Δ*pkac1* (red) were cultivated in Mandels-Andreotti medium supplemented with 1% (v/v) glycerol as the carbon source. Data points represent the mean dry biomass concentration (g/L) of three biological replicates. Dotted lines indicate exponential fits used to estimate the specific growth rate during the exponential phase. QM9414 showed a higher specific growth rate than Δ*pkac1* under these conditions.

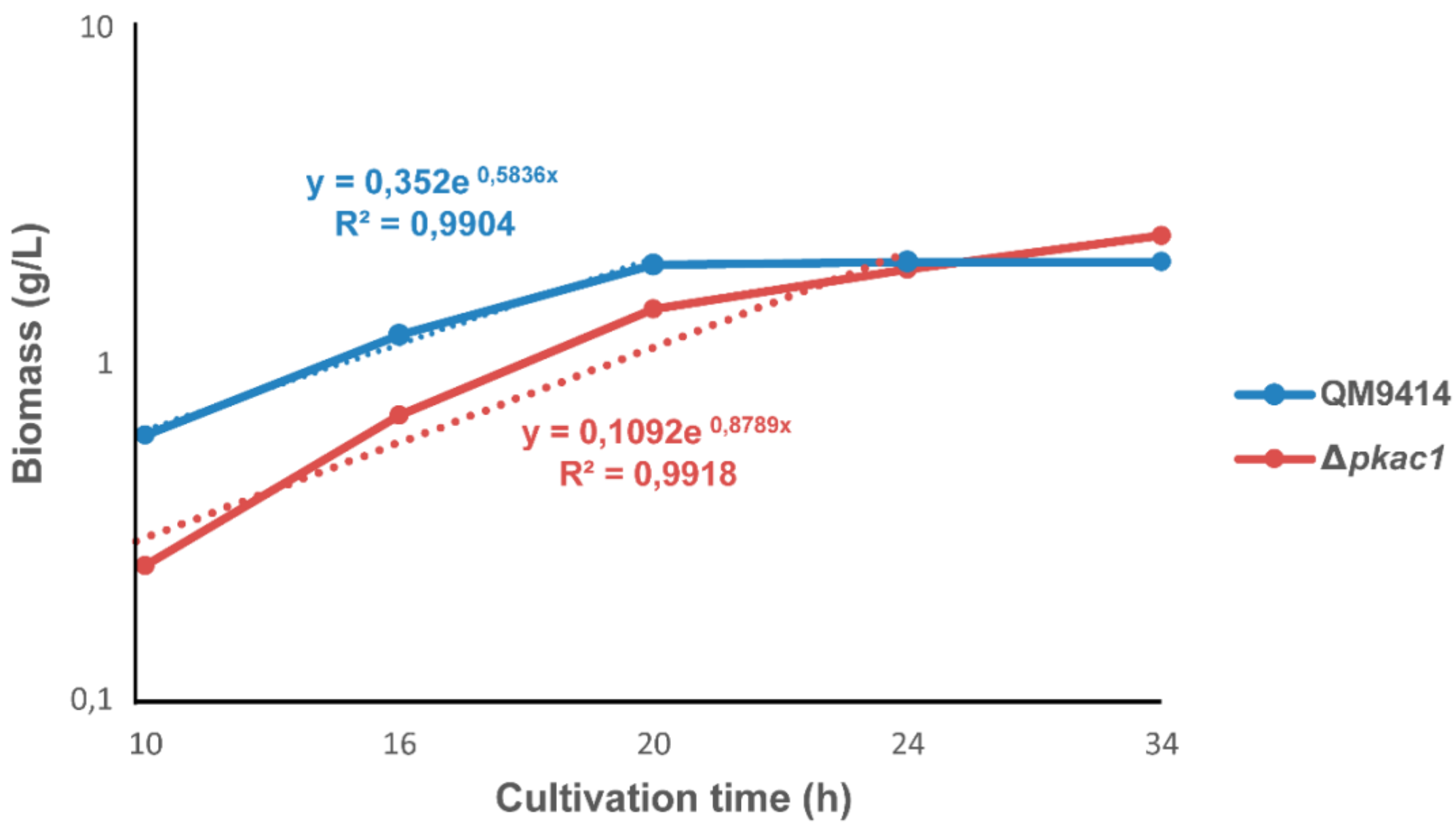

Online Resource 2. Phenotypic characterization of QM9414 and Δpkac1 strains. (a) Colony morphology on minimal media containing glucose, xylose, CMC, cellobiose, lactose, or glycerol (incubated for 4 days at 30 °C). Top row: Δ*pkac1*; Bottom row: QM9414. (b) Radial growth kinetics (0, 24, 96, 168 h) on the respective carbon sources; red circles = Δpkac1, blue squares = QM9414. Data points represent the mean ± SD (n = 3).

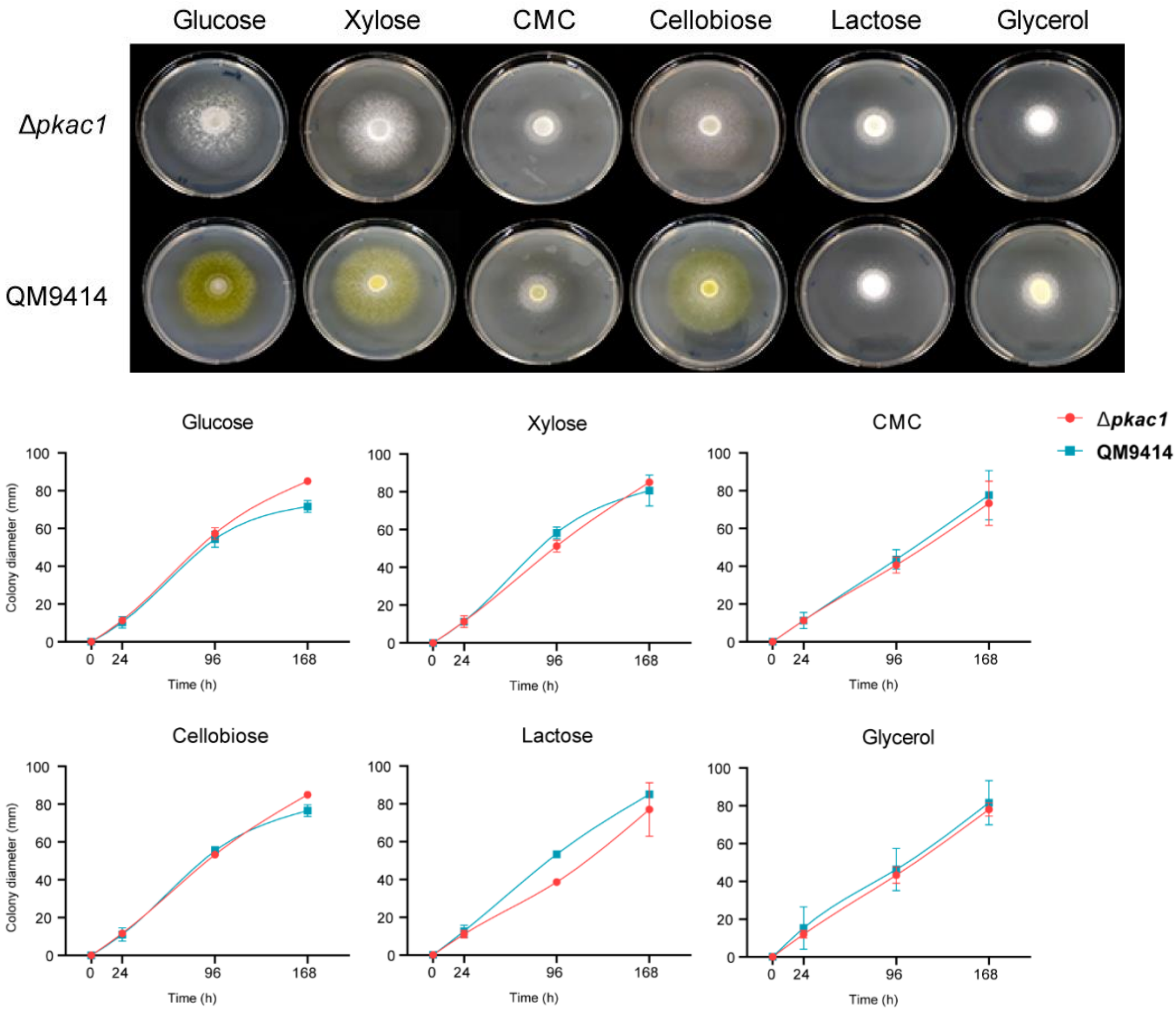

Online Resource 3. Principal Component Analysis (PCA) of the *T. reesei* transcriptome. The plot displays the first (PC1, 76% variance) and second (PC2, 12% variance) principal components. Each point represents an individual RNA-Seq library. Samples are color-coded by experimental group: QM9414 in glucose (red), Δ*pkac1* in glucose (green), QM9414 in sugarcane bagasse (blue), and Δ*pkac1* in sugarcane bagasse (purple). Ellipses indicate the 95% confidence interval for each group. The plot was generated in R using the *ggplot2* and *prcomp* packages.

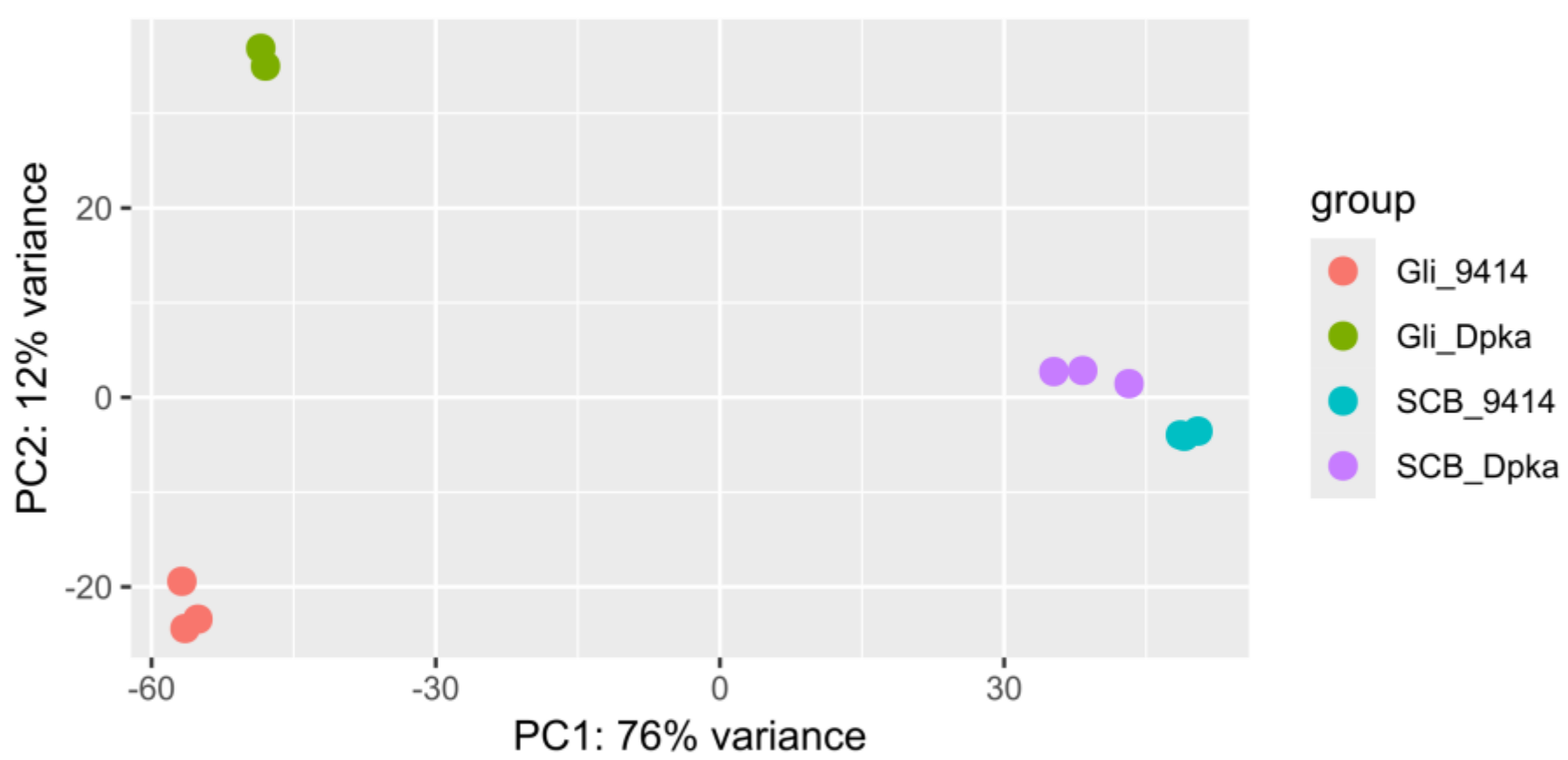

Online Resource 4. Volcano plots of differential gene expression. Each point represents a gene plotted according to its $\log_2$ fold change ($\log_2FC$, X-axis) and statistical significance ($-\log_{10}$ adjusted p-value, Y-axis). Differentially expressed genes (adjusted p-value ≤ 0.05 and $|\log_2FC| \geq 1$) are highlighted in red. The four comparisons displayed are: (a) Δ*pkac1* vs. QM9414 in glucose; (b) Δ*pkac1* vs. QM9414 in sugarcane bagasse; (c) bagasse vs. glucose in QM9414; (d) bagasse vs. glucose in Δ*pkac1*.

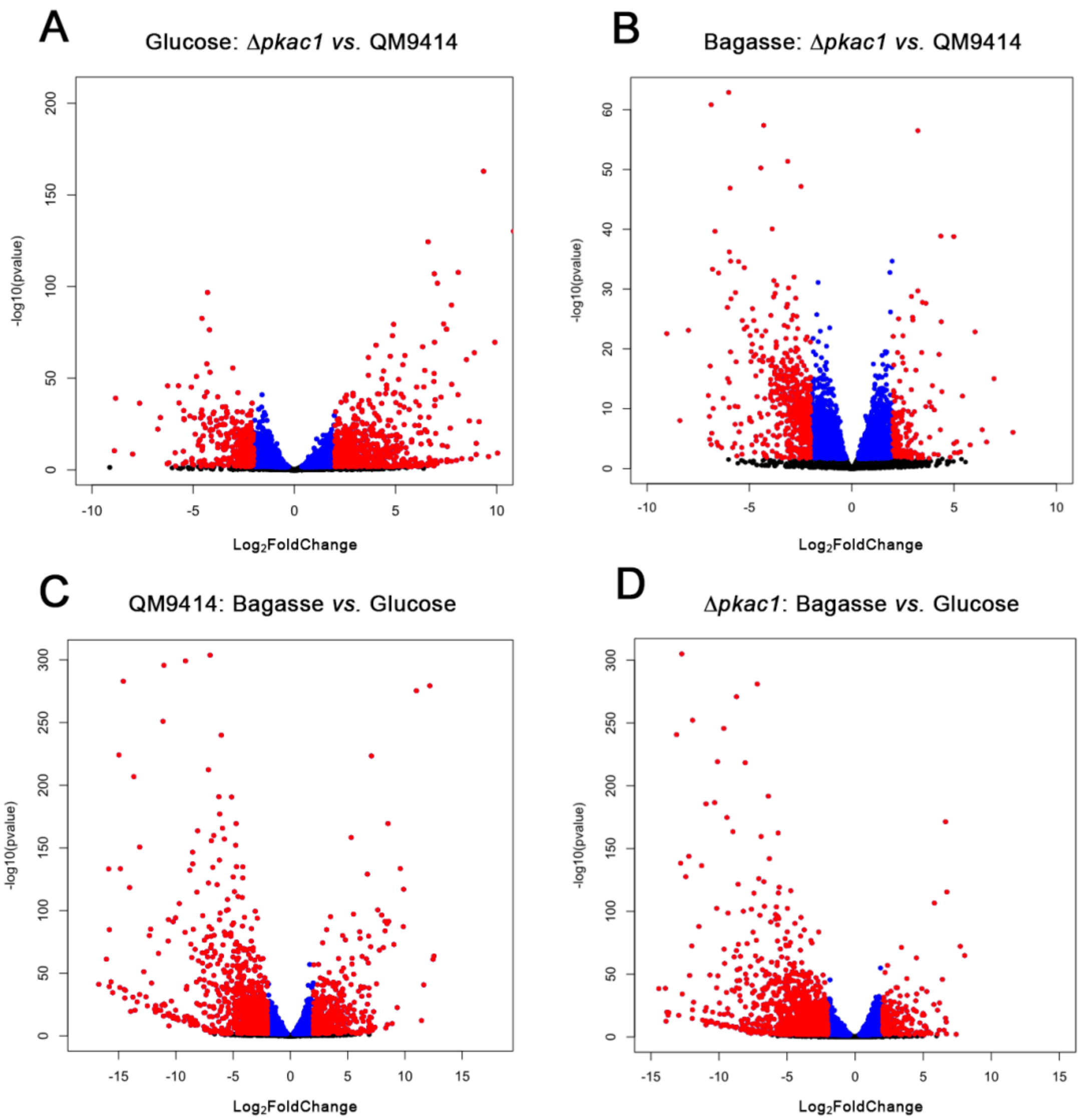

Online Resource 5. Venn diagrams illustrating the overlap of differentially expressed genes (DEGs) in QM9414 and Δ*pkac1* strains under sugarcane bagasse and glucose conditions. Values in parentheses denote the total number of DEGs for that specific strain-condition. Numbers within the circles (non-overlapping areas) correspond to strain-exclusive DEGs. Numbers in the overlapping regions indicate DEGs common to both strains under the same condition.

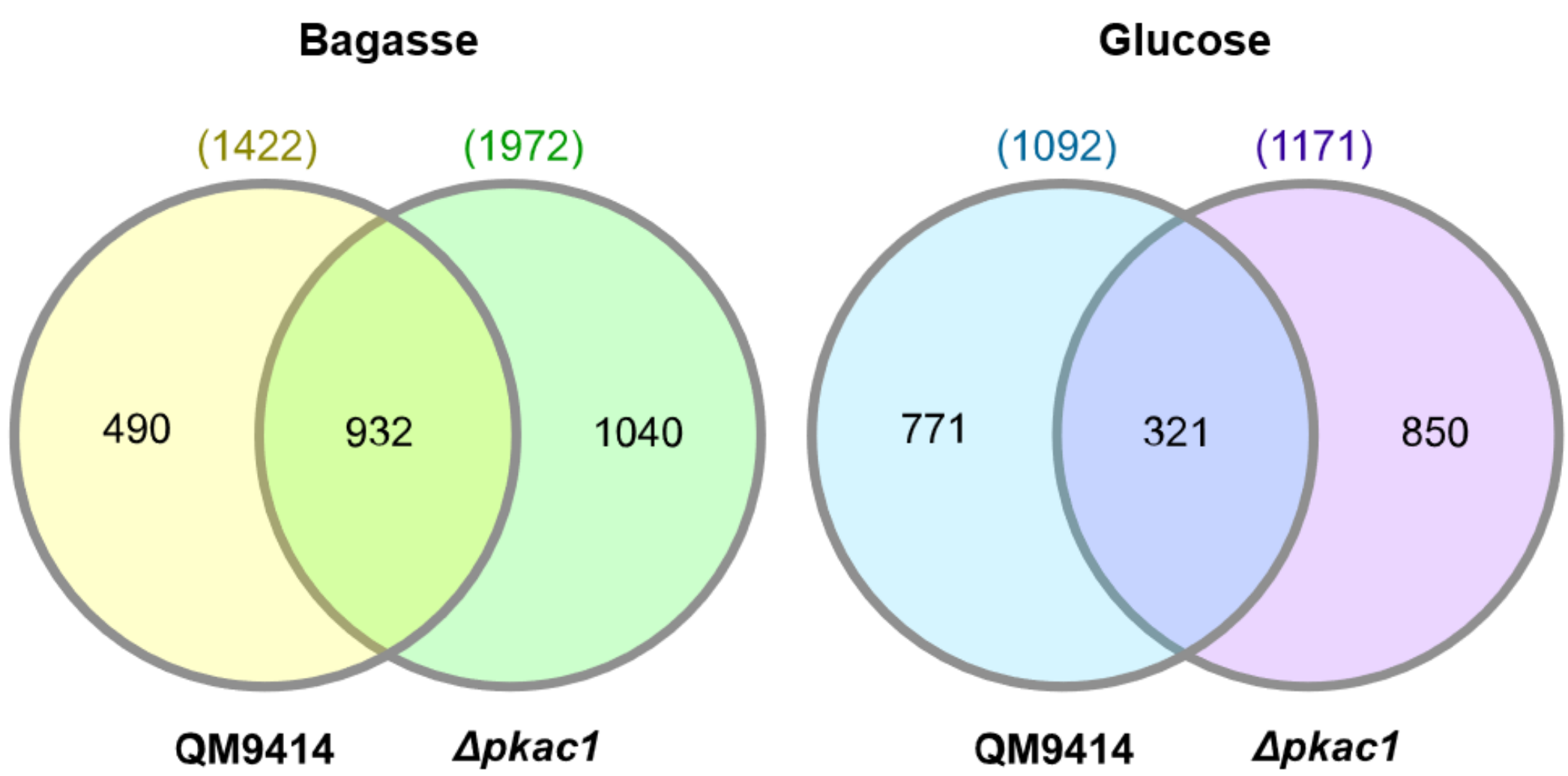

Online Resource 6. KEGG pathway functional enrichment of DEGs shared between the parental (QM9414) and mutant (Δ*pkac1*) strains of *T. reesei* cultivated in sugarcane bagasse. The lollipop chart displays major metabolic pathways associated with shared DEGs, including amino acid metabolism, energy metabolism, transport, biosynthesis of secondary metabolites, glutathione metabolism, and oxidative phosphorylation. Point color represents statistical significance (p-value), while point size indicates the number of genes annotated to the pathway. Line length corresponds to the degree of enrichment (Fold Enriched), and the Y-axis lists the most representative KEGG pathways. Only pathways with a p-value ≤ 0.05 are included.

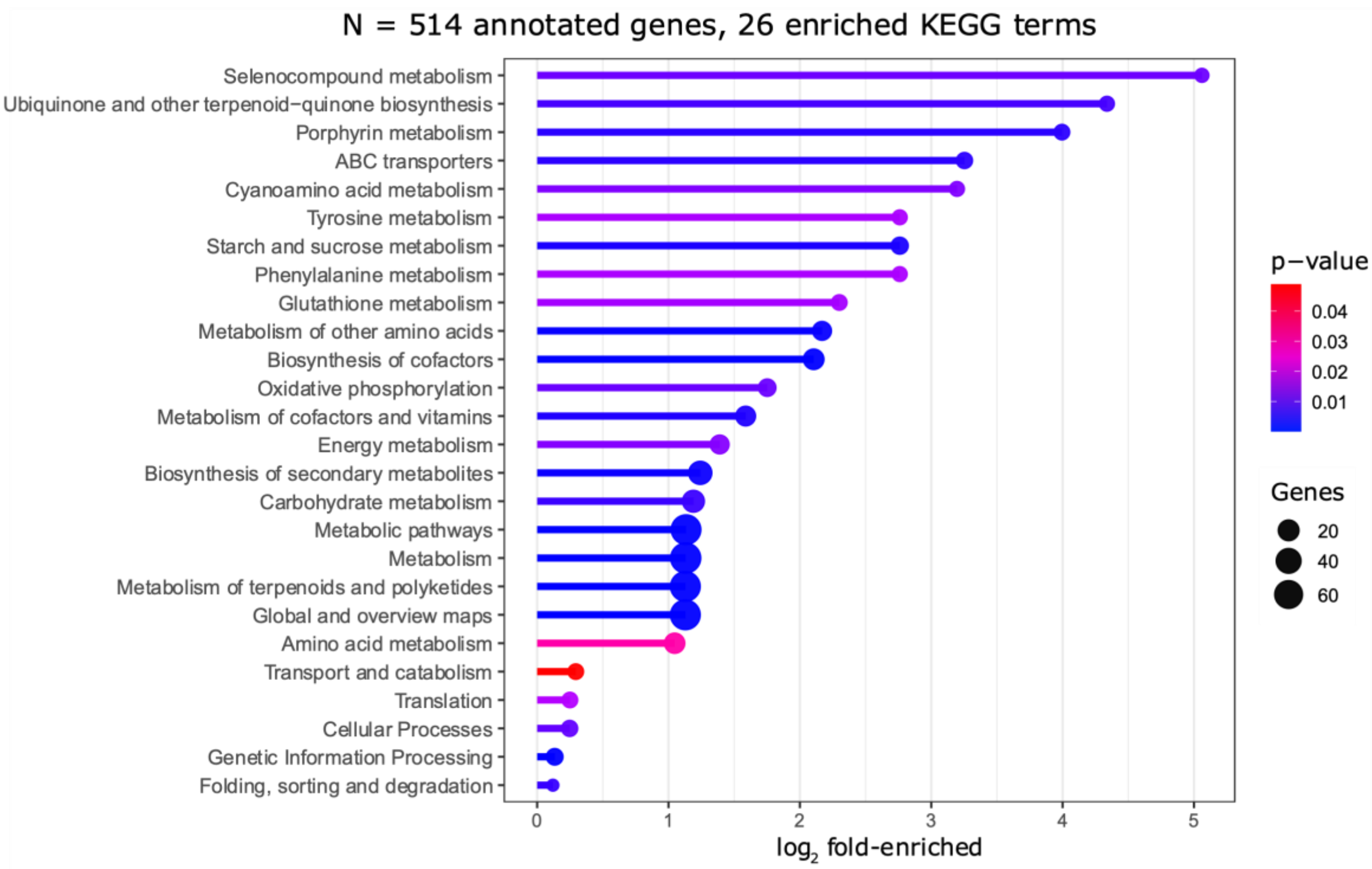

Online Resource 7. KEGG pathway enrichment analysis for shared differentially expressed genes in glucose. The chart shows significantly enriched metabolic pathways ($p \leq 0.05$) for DEGs common to both QM9414 and Δ*pkac1* strains in glucose medium. The representation follows the same scheme as Online Resource 6.

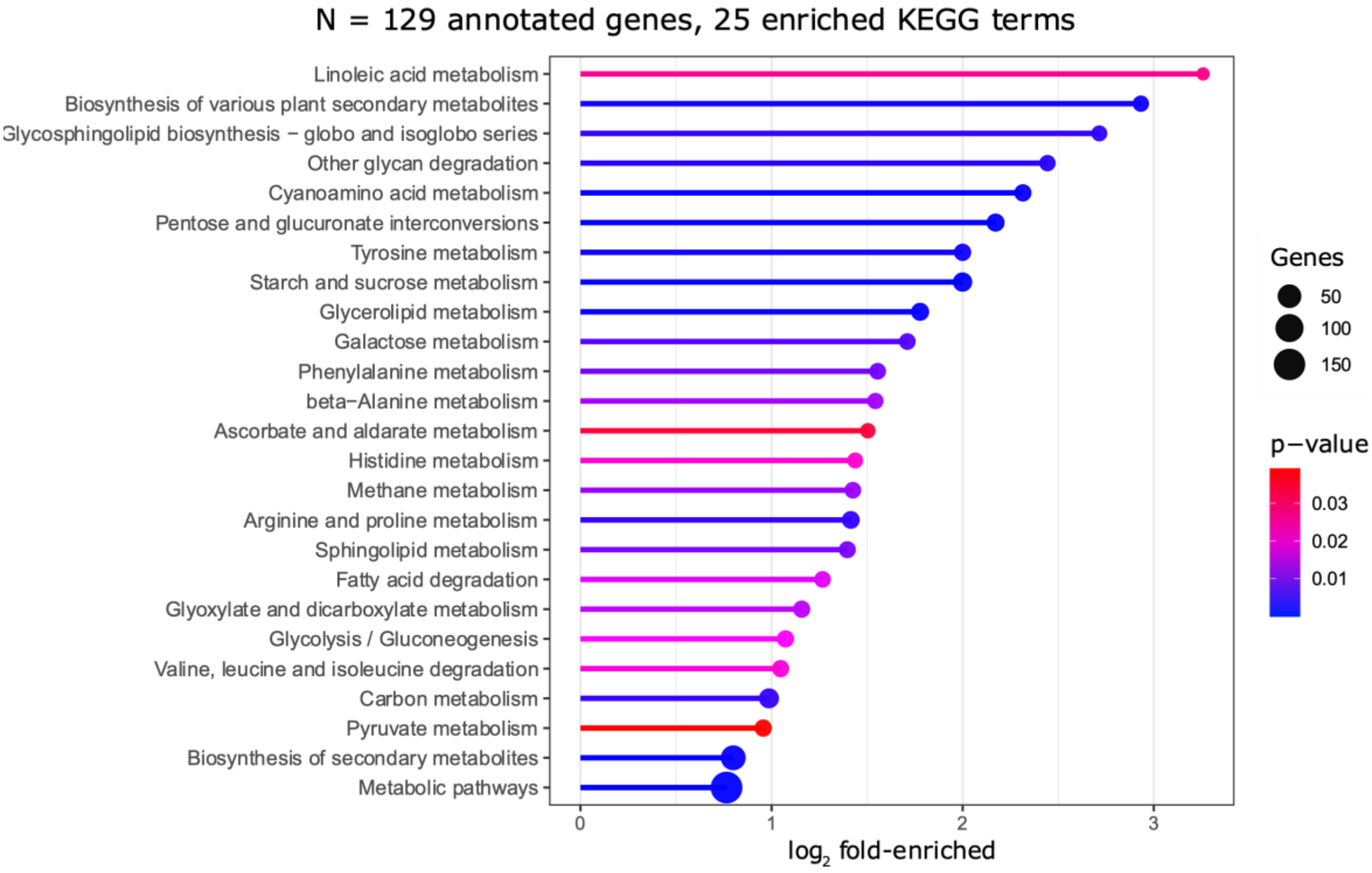

Online Resource 8. Functional distribution (KEGG) of strain-exclusive differentially expressed genes in response to sugarcane bagasse. Pie charts compare the distribution of functional categories for genes significantly regulated exclusively in the parental (QM9414) or mutant (Δ*pkac1*) strain when cultured in sugarcane bagasse relative to glucose. The categories represent major metabolic pathways and cellular processes, highlighting distinct transcriptional reprogramming between the two strains.

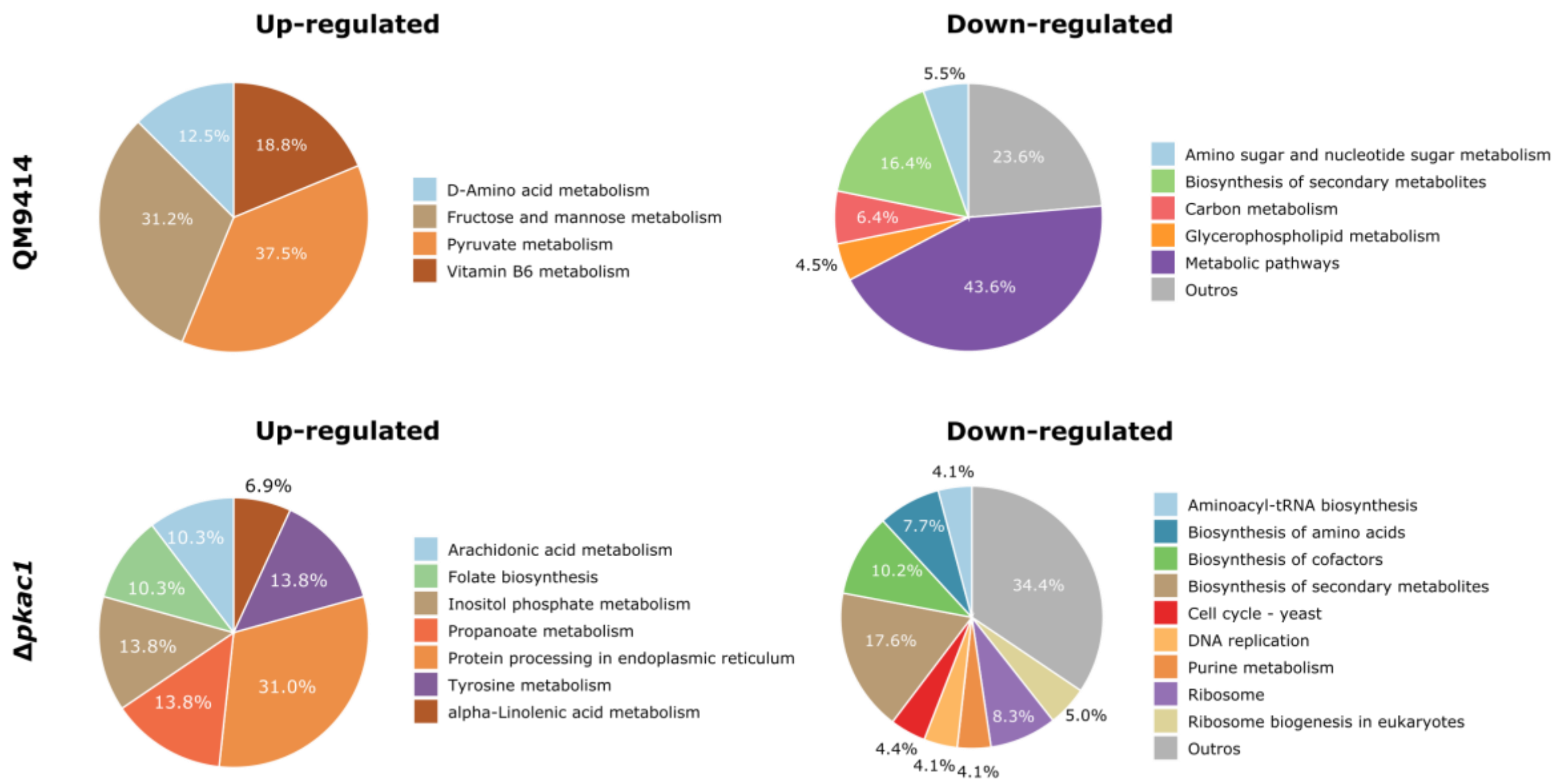

Online Resource 9. Hierarchical clustering heatmap of total protein relative abundance (total proteome) across culture conditions. Columns represent biological replicates for each condition. Colors indicate relative protein abundance (z-score), with green indicating lower abundance and red/brown indicating higher abundance. The lateral dendrogram clusters proteins with similar abundance profiles.

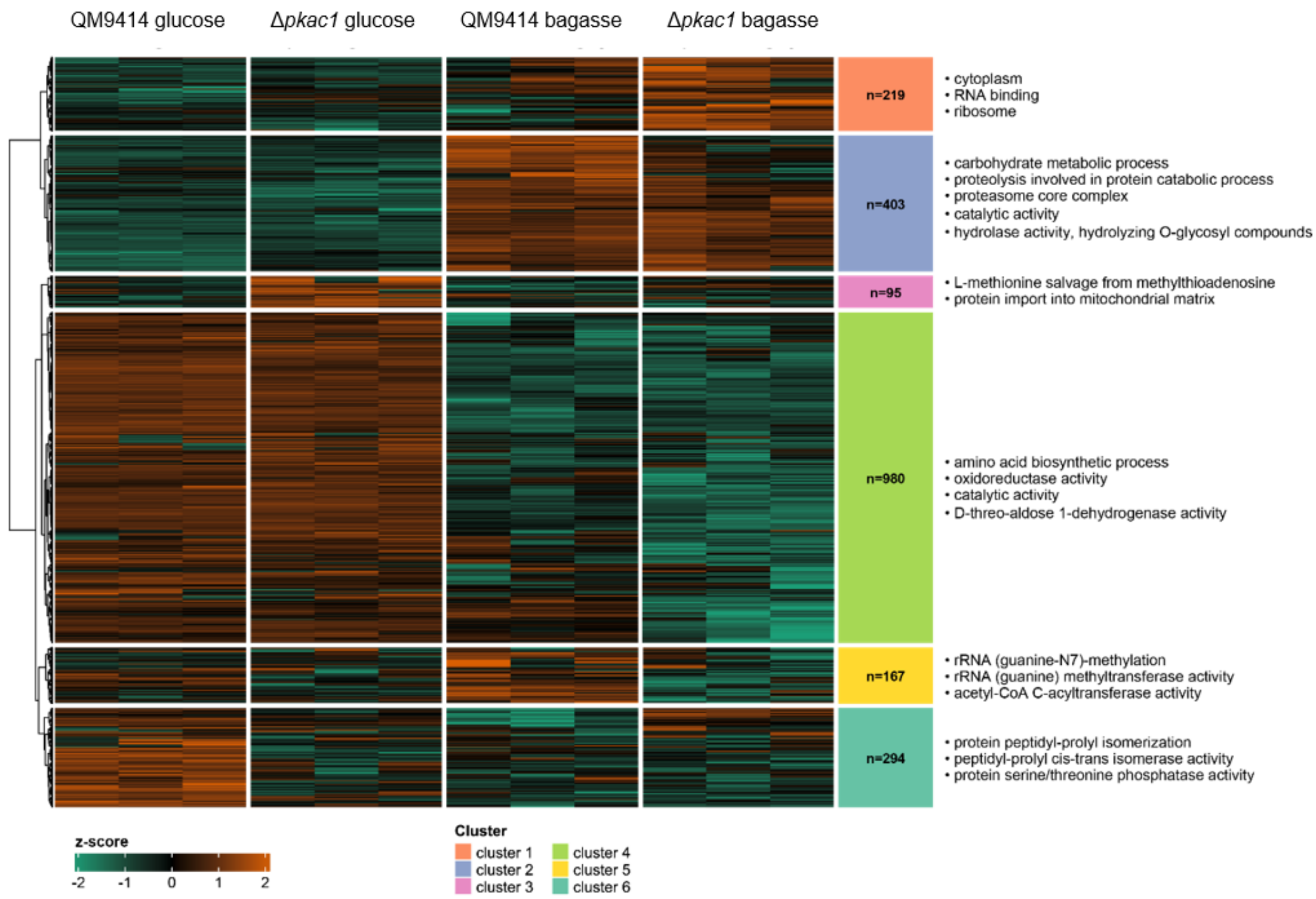

Online Resource 10. Volcano plots of differentially abundant proteins in the total proteome. Each point represents a protein plotted by abundance change ($\log_2$FC; Δ*pkac1* vs. QM9414, X-axis) and statistical significance (Y-axis). Proteins meeting the thresholds of $|\log_2 FC| \geq 1$ and FDR < 0.05 are shown as significantly different, and the most strongly changing proteins are labeled. (a) Sugarcane bagasse: Δ*pkac1* vs. QM9414. (b) Glucose: Δ*pkac1* vs. QM9414.

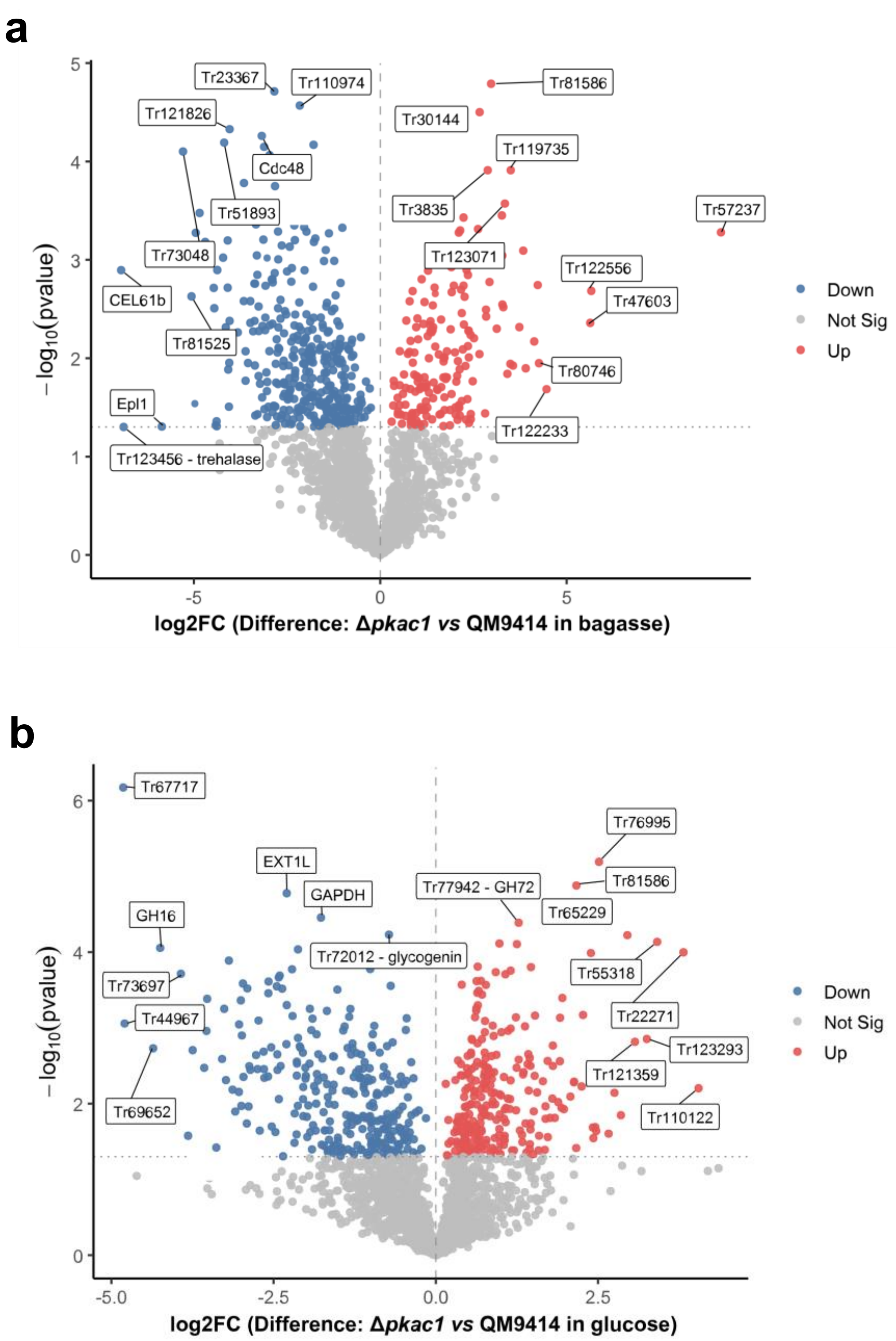

Online Resource 11. Phosphoproteomic landscape of *T. reesei* QM9414 and Δ*pkac1* in glucose and sugarcane bagasse. The heatmap groups phosphorylation sites based on their abundance profiles (z-score) across biological replicates for each condition. Rows represent phosphosites, and columns represent samples. Cluster analysis (right sidebar) reveals groups of sites with similar behavior and their respective most significant functional enrichments ($p \leq 0.05$).

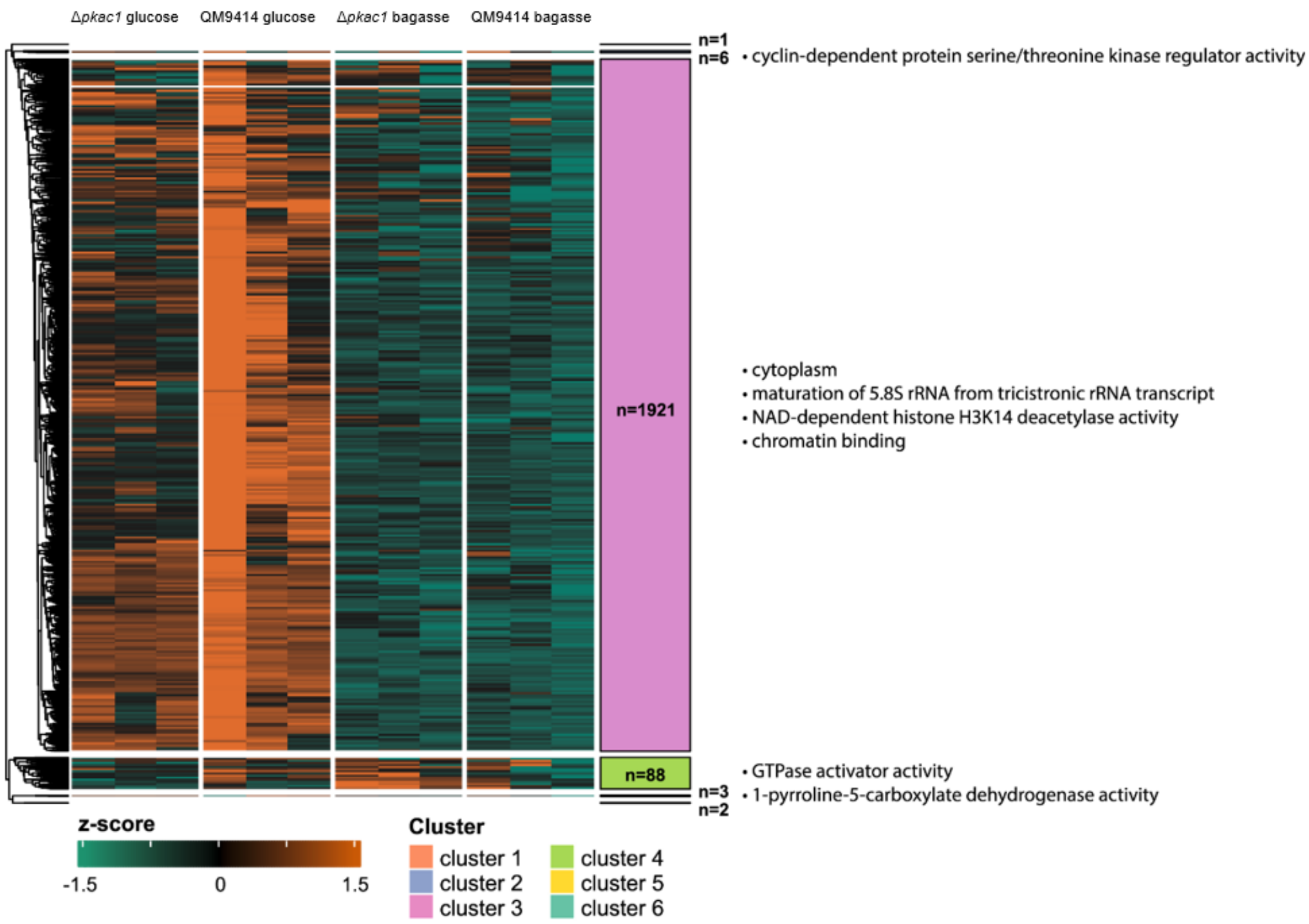

Online Resource 12. Differential phosphorylation analysis of candidate PKAc1-associated phosphoproteins. Volcano plots illustrate changes in the phosphorylation status of proteins containing the PKA consensus motif (R-R/K-x-S/T). The analysis compares the mutant (Δ*pkac1*) against the parental (QM9414) strain in (a) sugarcane bagasse and (b) glucose. The X-axis represents abundance change (log_2FC), and the Y-axis indicates statistical significance, with labels highlighting representative targets.

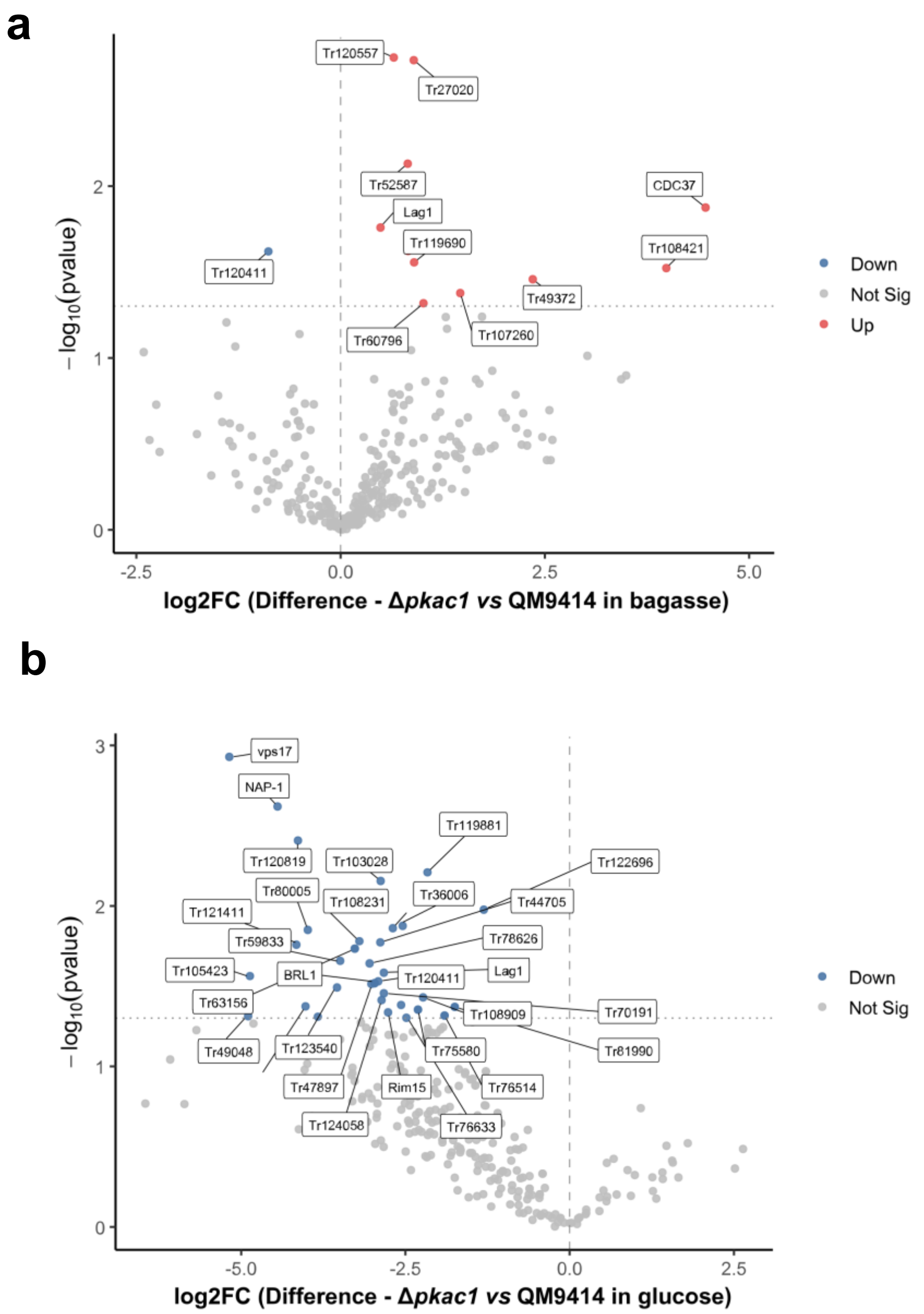

Online Resource 13. Quantitative overview of differential regulation across omics layers. Bar charts compare the number of up- and down-regulated elements in four experimental contrasts using cutoffs of $log_2FC \geq 1$ and $FDR \leq 0.05$. Each panel represents an analysis layer: differentially expressed transcripts (DEGs), differentially abundant proteins (DAPs), and phosphoproteins with differentially abundant phosphorylation sites (DAPhs).

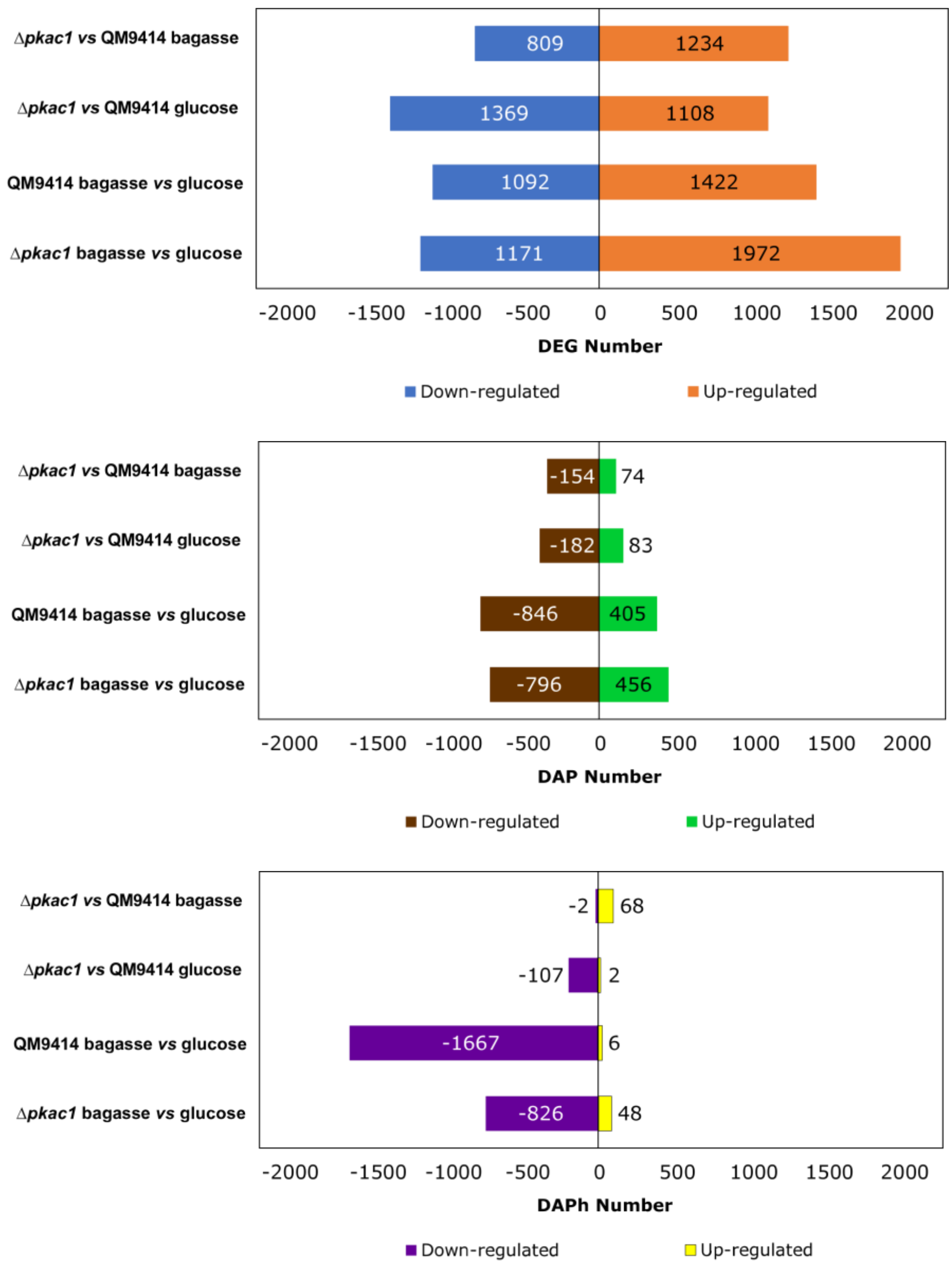

Online Resource 14. Top candidate PKAc1 substrates prioritized by phosphoproteomics. The table lists proteins exhibiting the most significant reduction in phosphorylation levels in the Δ*pkac1* strain that contain the PKA consensus motif.

Id	Contrast	Protein Position	Phosphorylated AA	Site Window	Score
Tr120411	Δ*pkac1* x QM9414 on Bagasse	517	S	HRRKSSTHH	0.872
Tr43877	Δ*pkac1* x QM9414 on Glucose	1824	S	KRRDSTTST	0.87
Tr120819	Δ*pkac1* x QM9414 on Glucose	206	S	VRRLSDDGA	0.854
Tr121214	Δ*pkac1* x QM9414 on Glucose	132	S	RRRYSEFAS	0.847
Tr76037	Δ*pkac1* x QM9414 on Glucose	448	S	LRRLSSSSF	0.839
Tr78626	Δ*pkac1* x QM9414 on Glucose	226	S	TRRESIVRR	0.713

Online Resource 15. Summary of peptide-PKAc1 docking simulations. The table summarizes simulation scores for each candidate peptide. "Score (rank1)" indicates the score of the pose with the best energy, and "ΔScore" represents the difference between this score and the PKAr1 control.

Protein Id	Score (rank1)	Top-5 Median	ΔScore
Tr76037	-224.523	-189.195	16.615
Tr120819	-220.784	-215.72	20.354
Tr120411	-216.953	-205.352	24.185
Tr43877	-208.246	-203.674	32.892
Tr121214	-198.642	-175.905	42.496
Tr78626	-198.49	-194.464	42.648
PKAr1	-241.138	-204.148	0

Online Resource 16. pKac1 gene deletion confirmation by PCR (A). RT-qPCR validation of selected transcriptomic targets and *pkac1* deletion status (B). Relative expression ($2^{-\Delta\Delta Ct}$) of *cel6a*, *cel7a*, *cre1*, *xyr1*, and *pkac1* in QM9414 and Δ*pkac1* cultivated in 1% glucose or 1% sugarcane bagasse for 24 h. Filled bars represent glucose-grown samples, and hatched bars represent sugarcane bagasse-grown samples. Values correspond to the mean ± SD of three biological replicates and were normalized using β-actin as the reference gene. Statistical significance was assessed using Student's *t* test: **** $p \leq 0.0001$; *** $p \leq 0.001$; ns, not significant. Detection of *pkac1* transcripts in QM9414 but not in Δ*pkac1* supports the deletion status of the mutant strain.

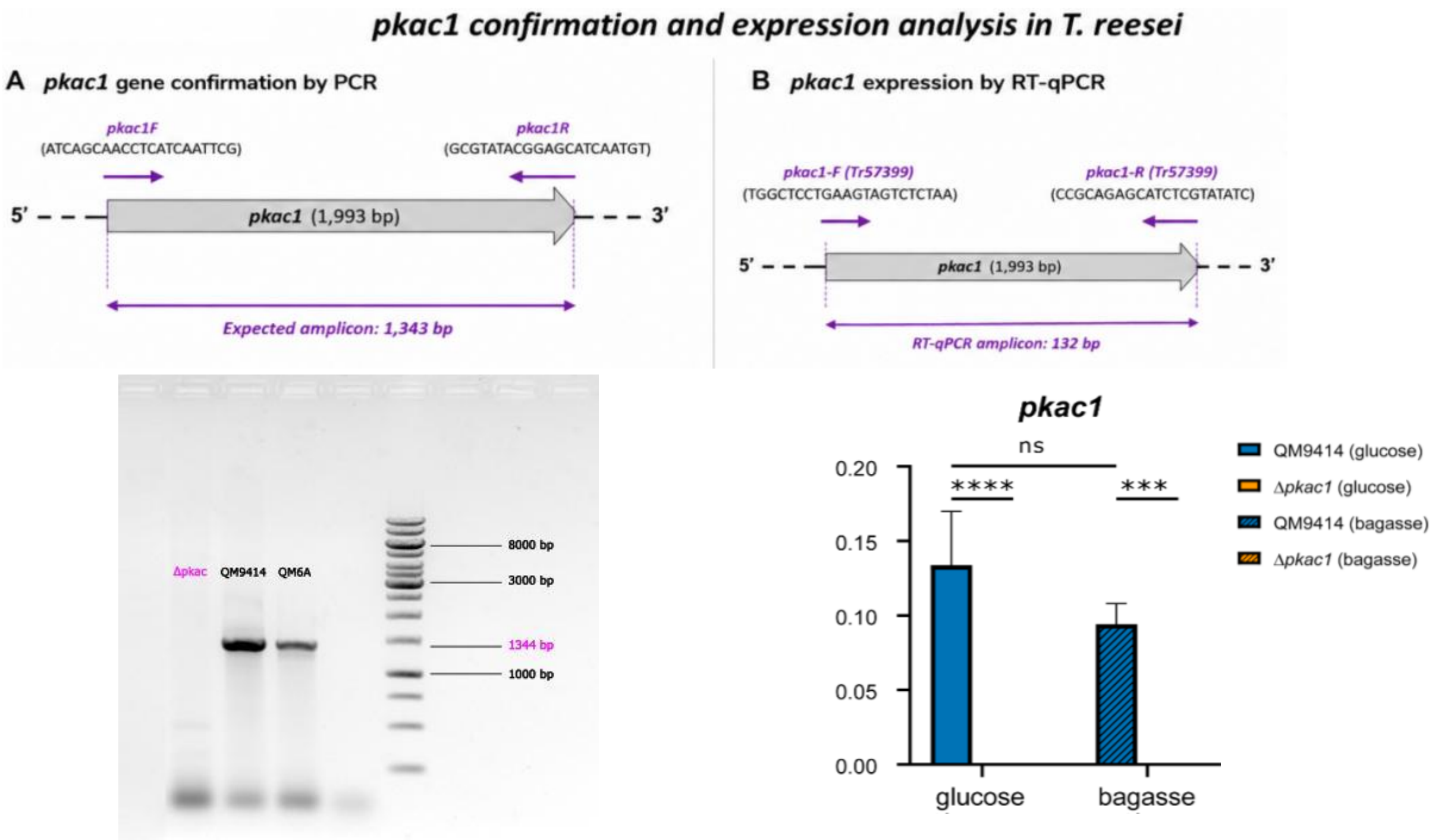

Supplement: Supplementary file 1 [file 11274_2026_5252_MOESM1_ESM.pdf]
